# Histone macroH2A1 is a stronger regulator of hippocampal transcription and memory than macroH2A2 in mice

**DOI:** 10.1038/s42003-022-03435-4

**Published:** 2022-05-19

**Authors:** Gurdeep Singh, Gilda Stefanelli, Klotilda Narkaj, Mark A. Brimble, Samantha D. Creighton, Timothy A. B. McLean, Meaghan Hall, Krista A. Mitchnick, Jacqueline Zakaria, Thanh Phung, Anas Reda, Amanda M. Leonetti, Ashley Monks, Lara Ianov, Boyer D. Winters, Brandon J. Walters, Andrew M. Davidoff, Jennifer A. Mitchell, Iva B. Zovkic

**Affiliations:** 1 Department of Cell & Systems Biology, University of Toronto, Toronto, ON M5S 3G3 Canada; 2 Department of Psychology, University of Toronto Mississauga, Mississauga, ON L5L 1C6 Canada; 3 Department of Surgery, St. Jude Children’s Research Hospital, Memphis, TN 38105 USA; 4 Department of Psychology, York University, Toronto, ON M4N 3M6 Canada; 5 Civitan International Research Center, University of Alabama at Birmingham, Birmingham, AL 35233 USA; 6 Department of Psychology, University of Guelph, Guelph, ON N1G 2W1 Canada; 7 Department of Biology, University of Toronto Mississauga, Mississauga, ON L5L 1C6 Canada

**Keywords:** Neuroscience, Chromatin

## Abstract

Histone variants H2A.Z and H3.3 are epigenetic regulators of memory, but roles of other variants are not well characterized. macroH2A (mH2A) is a structurally unique histone that contains a globular macrodomain connected to the histone region by an unstructured linker. Here we assessed if mH2A regulates memory and if this role varies for the two mH2A-encoding genes, *H2afy* (mH2A1) and *H2afy2* (mH2A2). We show that fear memory is impaired in mH2A1, but not in mH2A2-deficient mice, whereas both groups were impaired in a non-aversive spatial memory task. However, impairment was larger for mH2A1- deficient mice, indicating a preferential role for mH2A1 over mH2A2 in memory. Accordingly, mH2A1 depletion in the mouse hippocampus resulted in more extensive transcriptional de-repression compared to mH2A2 depletion. mH2A1-depleted mice failed to induce a normal transcriptional response to fear conditioning, suggesting that mH2A1 depletion impairs memory by altering transcription. Using chromatin immunoprecipitation (ChIP) sequencing, we found that both mH2A proteins are enriched on transcriptionally repressed genes, but only mH2A1 occupancy was dynamically modified during learning, displaying reduced occupancy on upregulated genes after training. These data identify mH2A as a regulator of memory and suggest that mH2A1 supports memory by repressing spurious transcription and promoting learning-induced transcriptional activation.

## Abstract (web summary)

The structurally-unique histone variant, mH2A1, influences learning and memory in mice to a greater extent than a similar variant, mH2A2.

## Introduction

Learning-induced changes in transcription and their regulation by epigenetic factors are necessary for establishing long-term memories^1^. Accordingly, dysregulation of epigenetic factors is implicated in age- and neurodegeneration-related cognitive deficits^2^, suggesting a critical role for epigenetic regulation in neural plasticity. Most research on the role of histones in memory has focused on post-translational modifications, but recent studies identified histone variants as epigenetic regulators of neural plasticity and memory formation^3–8^. Histone variants are non-allelic counterparts of the canonical histones H2A, H2B and H3 (H4 variants have not been identified in mammals) that differ from each other in structure and function^9^. In non-neuronal cells, the unique properties of histone variants result in distinct regulatory effects on transcription through variant-specific interactions with different partners and alterations of nucleosome stability^10^. In contrast to canonical histones, synthesis of histone variants is replication-independent and as such, histone variants become the primary source of histones in post-mitotic neurons, making them uniquely relevant for understanding the epigenetic basis of memory formation. Indeed, levels of the histone variants H3.3 and H2A.Z both increase in aged mice, with H3.3 becoming the dominant H3 histone in the adult brain^4,7^. Although a role for histone variants in the brain is only beginning to be studied, there is nevertheless evidence implicating histone-specific effects of H2A variants in neurodegeneration^11^. As such, uncovering the role of distinct histone variants in neural function will not only inform their role in memory, but also their function in non-dividing cells and their relevance as therapeutic targets.

We previously showed that the binding of histone H2A.Z, a variant of the canonical histone H2A, is dynamically modified during learning and that H2A.Z deficient mice have improved memory, suggesting that H2A.Z may be a memory suppressor^3–5,12,13^. Given that the H2A family of histones is particularly diverse and consists of several structurally distinct variants^9^, it stands to reason that different H2A variants may have unique functional roles in neural plasticity. MacroH2A (mH2A) is an especially strong candidate based on its unique structure among histones. In addition to its histone domain that shares 64% similarity with the canonical H2A, mH2A contains a large 30 kDa non-histone macrodomain on its C-terminal that makes it ~3 times larger than the canonical H2A^14^. This dramatic structural difference points to a potentially impactful role of mH2A in memory regulation.

mH2A is encoded by two genes, *H2afy* (encodes mH2A1) and *H2afy2* (encodes mH2A2), which produce unique protein products with the potential to further diversify mH2A function^15^. Both gene products are linked with repressive effects on transcription, but are also enriched on bivalent genes, indicating a potential role in stimulus-induced gene activation^16^. Moreover, both are implicated in cellular reprogramming, differentiation and development^17,18^, but they also have some unique functions in non-neuronal cells^19^. Indeed, *H2afy* has been selectively implicated as a risk factor and marker of disease progression in Huntington’s disease both in humans and mouse models^11^, suggesting a potential isoform-specific relevance of mH2A in neurodegeneration. Here, we conducted the first investigation of mH2A1 and mH2A2 function in the mouse hippocampus and its influence on memory formation. Using a combination of behavioural studies, virally-mediated gene depletion and genome-wide sequencing, we showed that loss of mH2A1 impaired memory and altered gene expression more than the loss of mH2A2, indicating a preferential role of mH2A1 in memory. This behavioural and transcriptional phenotype was associated with selective regulation of mH2A1 binding during learning, indicating that the histone variant mH2A has an isoform-specific role in regulating memory and transcription in the mouse brain.

## Results

### mH2A1 depletion preferentially regulates memory compared to mH2A2

**Fig. 1:**
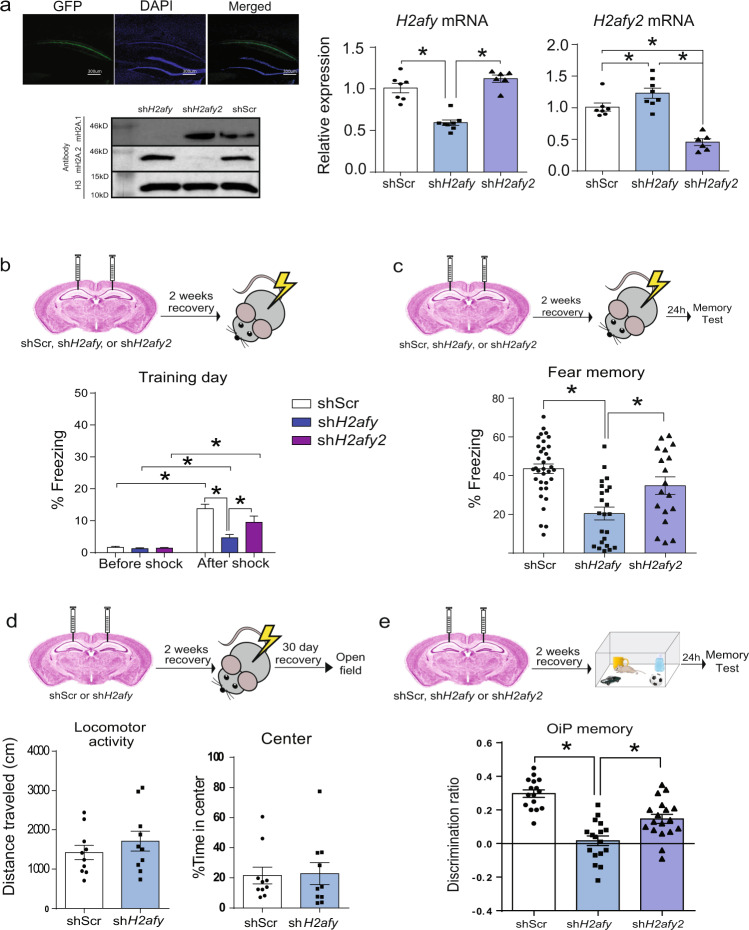
Hippocampal depletion of mH2A1 impairs memory more than the depletion of mH2A2. **a** Validation of mH2A1 (encoded by *H2afy* gene) and mH2A2 (encoded by *H2afy2* gene) depletion in the mouse hippocampus. **b** During the fear memory training session, all mice show increased freezing after receiving shock, suggesting that learning has occurred in all groups. **c** Depletion of mH2A1, but not of mH2A2, impairs recall of fear memory 24 h after training (N: shScr = 35, sh*H2afy* = 24, sh*H2afy2* = 18). **d** mH2A1 depletion does not affect locomotor activity (*N* = 10/group). **e** mH2A1 and mH2A2 depletion each impair memory for Object in Place, but the impairment is greater in mH2A-1 than in mH2A2-depleted mice (n: shScr = 16, sh*H2afy* = 17, sh*H2afy2* = 18). Data are shown as mean ± SEM. (ns *p* > 0.05, *p < 0.05).

To evaluate if mH2A1 and mH2A2 regulate memory, we depleted each gene in area CA1 of the hippocampus, a brain region critical for memory. Specifically, mH2A1- (*H2afy*) or mH2A2- (*H2afy2*) targeted shRNAs were packaged in adeno-associated viral vectors pseudotyped with DJ capsid (AAV-DJ). The vectors were delivered directly into dorsal area CA1 using stereotaxic surgery and knockdown was confirmed in the infected tissue. Each shRNA selectively depleted the intended mH2A target (i.e., mH2A1 or mH2A2) at both protein (Fig. 1a and Supplementary Fig. 1) and mRNA levels (Fig. 1a). Indeed, anti-*H2afy* shRNA treatment selectively reduced *H2afy* (encodes mH2A1) mRNA without influencing *H2afy2* (encodes mH2A2) expression (*F*_2,19_ = 13.28, *p* < 0.001). Similarly, anti-*H2afy2* shRNA treatment resulted in reduced *H2afy2* expression (*F*_2,19_ = 28.43, *p* < 0.0001), although an increase in *H2afy* mRNA was also observed (Supplementary Data 1).

To assess if mH2A1 and mH2A2 regulate memory, mice in each condition (scramble control, shRNA against *H2afy* or *H2afy2*) were trained with contextual fear conditioning, a task in which mice learn to associate a novel context with exposure to a foot shock. Training involved 2 min of exploration of the novel context, followed by 1 foot shock (0.5 mA, 2 sec long) and one additional minute of exploration before returning to the home cage. During training, there was no difference in exploration before shock exposure, as indexed by similarly low levels of freezing across groups. After shock, sh*H2afy* mice froze less than shScr (*p* < 0.001) and sh*H2afy2* (*p* = 0.04) mice (Fig. 1b), but all groups (all *p* < 0.05) showed increased freezing (index of fear memory) after shock than before shock [Shock (Pre, Post) × Virus (shScr, sh*H2afy*, sh*H2afy2* interaction: *F*_2,74_ = 9.52, *p* < 0.001)], suggesting that shock exposure induced learning in all groups during the training session (Supplementary Data 2).

Fear memory was assessed 24 h after training by measuring the amount of freezing behaviour upon re-exposure to the training context in the absence of shock, where higher levels of freezing indicate stronger memory. We observed a deficit in freezing behaviour only in mH2A1-depleted mice compared to all other groups (*F*_2,74_ = 15.25, *p* < 0.001; all post-hocs *p* < 0.05). In contrast, mH2A2 depletion had no effect on freezing (shScr vs sh*H2afy2*: *p* = 0.07), suggesting that this histone is not critical for fear memory (Fig. 1c and Supplementary data 2). Given that fear memory is assessed by the amount of time mice spend freezing, we conducted additional testing in an open field (OF) to exclude potential differences in motor activity as contributors to the fear memory deficit in mH2A1-depleted mice. Using this approach, we found no differences in activity levels or the amount of time spent in the centre (more centre time suggests less anxiety) of the OF in mH2A1-depleted mice compared to scramble controls, suggesting that reduced freezing is not a reflection of hyperactivity in this group (Fig. 1d and Supplementary data 3). Although these mice were tested for locomotor activity 30 days after fear conditioning, *H2afy* expression was still reduced at this time point (*t*_8_ = 5.51, *p* = 0.001; Supplementary Fig. 2), suggesting that similar performance between mH2A1-depleted and control mice in the OF was not attributable to reduced efficacy of the AAV over time.

Fear conditioning is a highly robust memory paradigm that may mask a potential subtle effect of mH2A2 depletion on memory. To test this possibility, a separate cohort of mice was tested using a less robust and non-aversive memory paradigm, the Object-in-Place (OiP) test. OiP is a hippocampus-dependent memory test in which the location of 2 out of 4 familiar objects is switched during testing, such that mice utilise spatial memory without relying on an aversive stimulus^20^. Using this test, we found that both mH2A1- (*p* < 0.0001) and mH2A2- (*p* < 0.0001) deficient mice had impaired ability to discriminate the switched objects (discrimination ratio) compared to scramble controls (*F*_2,48_ = 27.05, *p* < 0.001). Moreover, mH2A1-depleted mice were more impaired than mH2A2-depleted mice (*p* = 0.001), indicating that mH2A2 does have some impact on object location memory (Fig. 1e and Supplementary Data 4), whereas mH2A1 depletion consistently results in a robust memory deficit, irrespective of the task.

Given that OiP was the most sensitive measure in its ability to detect a functional effect of mH2A2, we conducted additional validation of this phenotype using a new set of shRNA constructs against each mH2A-encoding gene to ensure specificity of the effect. With the new constructs packed in the same AAV vector, we found that mH2A1 (*p* < 0.0001) and mH2A2 (*p* = 0.05) depleted mice again had impaired OiP memory compared to scramble control (*p* < 0.0001) (*F*_2,26_ = 13.85, *p* < 0.0001) mice. Similarly, mH2A1 depletion again produced greater memory impairment than mH2A2 depletion (*p* = 0.005), thus reinforcing the validity of our findings (Supplementary Fig. 3).

### mH2A1 is a more potent repressor of basal hippocampal transcription than mH2A2

**Fig. 2:**
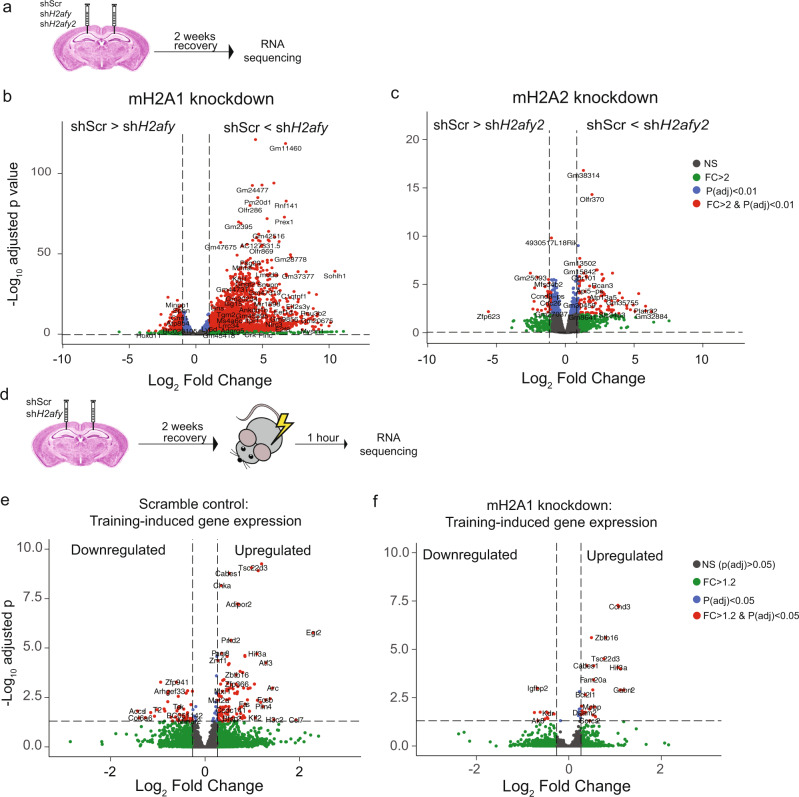
mH2A1 depletion promotes basal gene expression and blocks learning-induced gene induction. **a** Experimental design. Mice underwent stereotaxic surgery for intra-hippocampal (CA1) injections of AAV-DJ vectors carrying shRNA constructs against a scramble control sequence, *H2afy* or *H2afy2*. After 2 weeks of recovery, the infected region was extracted and RNA was sequenced. **b** Intra-CA1 depletion of *H2afy* (encodes mH2A1) alters the hippocampal expression of 2350 genes, majority of which (85%) were upregulated (significance cut-off: p adj < 0.01 and FC > 2). **c** Intra-CA1 depletion of *H2afy2* (encodes mH2A2) alters the expression of 127 genes, 69% of which were upregulated (significance cut-off: p adj < 0.01 and FC > 2). **d** Experimental design for testing the effects of mH2A1 depletion on training-induced gene expression. Mice underwent stereotaxic surgery for intra-CA1 injections of shScr or sh*H2afy* and were given 2 weeks to recover. After 2 weeks, half of the mice were fear conditioned and half were left undisturbed in their home cage. RNA was extracted from infected tissue and gene expression was compared in trained vs untrained mice for each virus. **e** In area CA1 of the hippocampus of scramble control mice, fear conditioning altered the expression of 169 genes, 122 of which were upregulated (significance cut-off: p adj < 0.05 and FC > 1.2). **f** In area CA1 of the hippocampus of mH2A1-depleted mice, fear conditioning altered the expression of 44 genes, 33 of which were upregulated (significance cut-off: p adj < 0.05 and FC > 1.2). *n* = 3 mice/group.

Having established a preferential role for mH2A1 over mH2A2 in memory, we examined whether these histones have distinct effects on transcription in hippocampal area CA1. mH2A1 or mH2A2 were virally depleted and the infected tissue was collected for RNA sequencing 2 weeks later (Fig. 2a). At a stringent significance cut-off (p adj < 0.01 and fold change > 2), mH2A1 depletion resulted in 2350 DEGs, of which 85% were up-regulated, suggesting that mH2A1 has a primarily repressive role in hippocampal transcription (Fig. 2b and Supplementary Data 6). In contrast, mH2A2 knockdown altered the expression of only 127 genes (Fig. 2c), ~18-fold fewer than mH2A1 depletion, with 69% of these DEGs being upregulated. A comparison of genes that were affected by mH2A1 compared to mH2A2 depletion shows that over half (89/127; 70%) of the DEGs altered by mH2A2 are also altered by mH2A1 (*p* < 0.0001, hypergeometric test) and most were upregulated in both conditions (Supplementary Tables 1–3). Thus, both mH2A histones have a repressive transcriptional effect in the hippocampus, but the broader loss of gene repression with mH2A1 depletion over mH2A2 depletion is consistent with its larger impact on memory formation.

We used EnrichR^21,22^ gene ontology analyses to evaluate the functional relevance of genes that were affected by mH2A1 depletion (Supplementary Fig. 3b). Upregulated DEGs produced largely overlapping categories that included terms such as lysosomal lumen, secretory granule lumen, vacuolar lumen and secretory granule lumen, each of which contains genes that are important for breaking down proteins and cellular debris. These upregulated pathways are likely detrimental for neural function as many neurodegenerative diseases are associated with lysosomal dysfunction and vesicle defects^23^. This is especially notable because lysosomal dysfunction is an important contributor to Huntington’s disease^24^ and mH2A1 has been identified as a biomarker that tracks Huntington’s disease severity in human patients and mouse models^11^. An inspection of the top 40 classified genes identified several with important roles in synaptic function, such as the vesicle-associated membrane protein 8 (*Vamp8*), its interacting partner *Snap23* (synaptosomal associated protein 23), as well as integrins (*Itgav*) and disintegrins (*Adam8*), suggesting that dysregulation of synaptic proteins may contribute to the observed memory deficits.

For downregulated DEGs, the analysis identified well known regulators of neural plasticity that are localised to dendrites, such as reelin (Reln), several GABA receptor subunits (GABRG1 and 3, GABRD), voltage-gated potassium channels (*Kcnab1*,*Kcnc2, Kcnd2*), as well as the Shank family of synaptic proteins (*Shank2*) (Supplementary Fig. 3c). The fact that mH2A1 depletion resulted in the reduced expression of many genes that encode dendritic proteins suggests that the loss of mH2A1 may be detrimental for neural plasticity by impacting synaptic function.

### mH2A1 depletion blocks learning-induced gene expression

Learning-induced changes in gene expression are necessary for establishing long-lasting memories, and dysregulation of learning-induced transcription impairs memory formation^1^. Given the extensive upregulation of gene expression under baseline conditions in mH2A1-deficient mice, we conducted additional RNA sequencing to determine if impaired memory is also associated with altered transcriptional induction during learning. Learning-induced gene expression in area CA1 of the hippocampus was assessed by comparing transcript levels in fear conditioned (1 h after training) mice to untrained controls either in the scramble control condition, or in mice with mH2A1 depletion (Fig. 2d). In scramble control mice, fear conditioning altered the expression of 169 genes (122 were upregulated) (Fig. 2e and Supplementary Data 8), whereas only 44 genes (33 were upregulated) were altered in mH2A1-deficient mice (Fig. 2f). Thus, learning induced 75% fewer DEGs in mH2A1-deficient mice compared to scramble controls (p adj < 0.05), indicating a strongly blunted learning response. Indeed, mH2A1 depletion blocked the induction of key memory-related genes, including *Arc, Fos, FosB, Egr1* and *Egr2*, which were upregulated by training in scramble controls, but were not affected in mH2A1-deficient mice. Together, these data indicate that mH2A1 is a powerful repressor of steady-state transcription, whereby its virally-mediated depletion leads to extensive upregulation of basal transcription and impaired transcriptional induction during learning.

### mH2A1 and mH2A2 binding is negatively associated with transcription in area CA1 of the hippocampus

**Fig. 3:**
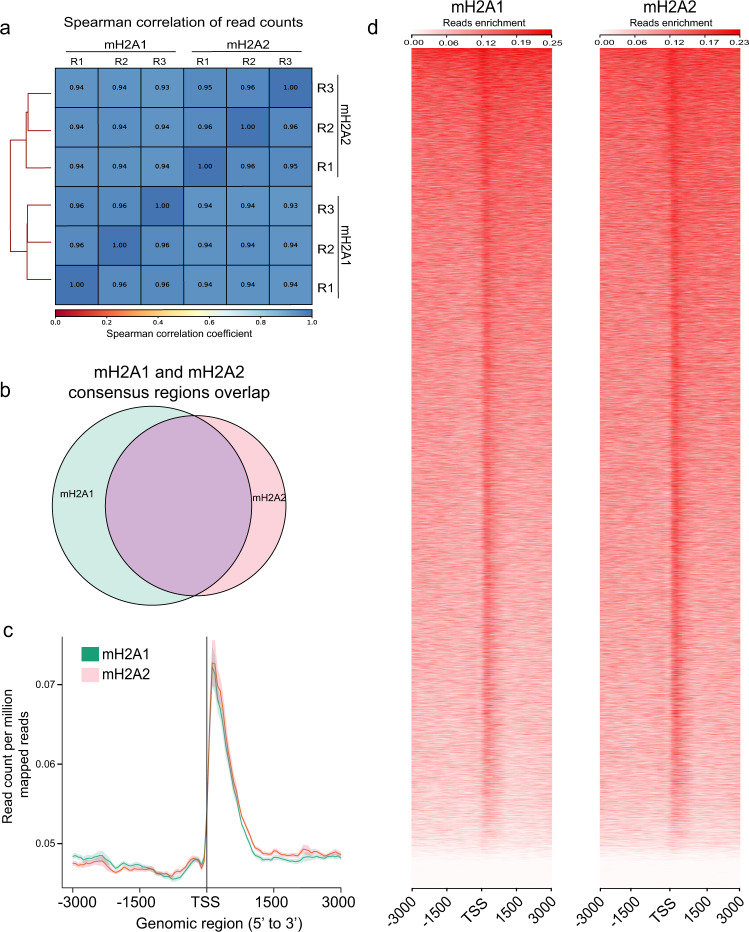
Characterisation of mH2A1 and mH2A2 binding in area CA2 of the hippocampus. **a** Correlation analysis of mH2A2 and mH2A2 binding for each of the three experimental replicates (R1-R3) of untrained control mice shows that mH2A1 and mH2A2 binding is strongly related. **b** Venn diagrams demonstrate the high level of overlap between mH2A1 and mH2A2 regions in untrained mice. **c** Average profile plot of mH2A1 and mH2A2 signal at TSS-flanking regions. **d** Heatmap of mH2A1 (left) and mH2A2 (right) enrichment at TSS-flaking regions. Heatmap is relating TSS regions ranked based on mH2A1 and mH2A2 signal.

Our data indicate that mH2A1 is a transcriptional repressor whose loss impairs memory more extensively than mH2A2, leading us to investigate potential differences in their chromatin occupancy and learning-induced dynamics. Using chromatin immunoprecipitation sequencing (ChIP-seq) against mH2A1 and mH2A2 in area CA1, we observed a high degree of overlap between mH2A1 and mH2A2 binding genome-wide (Fig. 3a, b) and around gene transcription start sites (TSS) (Fig. 3c, d). These data are consistent with a high degree of overlap between the two proteins in non-neuronal cells^17,25^, suggesting that their binding patters are similar.

In non-neuronal cells, mH2A can occur as narrow peaks, as well as broad domains that can span 200 kB regions^16,17^, with evidence that broad domains predominate in differentiated cells^26^. To assess the different types of mH2A signal in the brain, we used epic2 to detect broad and diffusely enriched domains, and MACS2 to detect comparatively narrower peaks (<1 kb)^27,28^. On average per sample, epic2 identified 77,705 broad domains (average length = 5590 bp) for mH2A1 and 59,247 broad domains for mH2A2, whereas MACS2 identified 279,596 peaks (average length = 347 bp) for mH2A1 and 129,496 peaks for mH2A2. Compared to a study that conducted mH2A1 and mH2A2 ChIP-seq in dermal fibroblasts and used both approaches to identify broad domains and narrow peaks, our data produced a greater number of broad domains (77,705 here vs 32,339 in dermal fibroblasts) and peaks (279,596 here vs 156,296 in dermal fibroblasts) for mH2A1. In contrast, the number of mH2A2 peaks were more similar to dermal fibroblasts for broad domains (59,247 here *vs* 55,751 in dermal fibroblasts) and slightly lower for narrow peaks (129,496 here *vs* 165,617 in dermal fibroblasts)^17^. Thus, our data demonstrate that mH2A in the brain occupies both broad domains and narrow peaks.

**Fig. 4:**
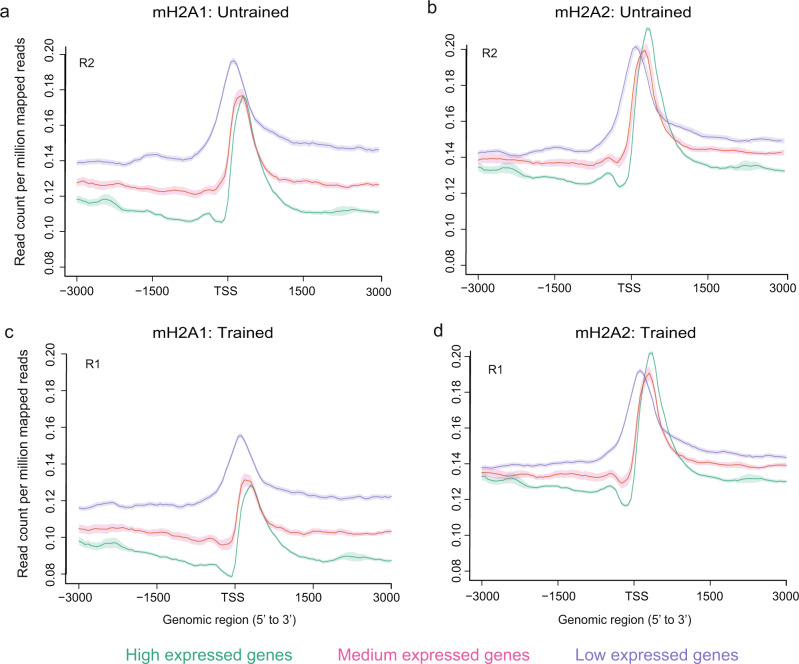
mH2A1 and mH2A2 binding is negatively associated with gene expression. Average profile plot of mH2A1 binding at the TSS for all genes grouped into three categories of gene expression (high, medium and low) shows that mH2A1 (**a**) and mH2A2 (**b**) enrichment is inversely proportional to gene expression in untrained (**c**) and trained (**d**) mice. Data for each mH2A protein are shown for individual replicates from untrained (replicate 2; R2) and trained (replicate 1; R1) groups.

Extensive transcriptional upregulation in the hippocampus of mH2A1-deficient mice, and to a lesser degree in mH2A2-deficient mice, suggests that mH2A histones play a primarily repressive function in the brain, as they do in other tissues^29^. To investigate if this relationship is evident in chromatin, we sorted RNA sequencing data based on gene expression levels (high, medium and low; see Methods section) and confirmed that the most highly expressed genes have the lowest levels of mH2A binding, and genes with lowest expression have the highest mH2A binding (Fig. 4 and Supplementary Fig. 4). This effect did not depend on learning, as both trained and untrained mice exhibited an inverse relationship between mH2A binding and gene expression. Though the distinction between high and medium gene expression categories was similar for mH2A1 and mH2A2 within the 2 kb region upstream of the transcription start site (TSS) (*p* < 0.0001, ANOVA), the distinction between high and low expressed genes for the 500 bp region upstream of the TSS was stronger for mH2A1 (*p* < 0.0001, ANOVA) than for mH2A2 (*p* < 0.01, ANOVA), indicating that mH2A1 might be a dominant repressive mark in the hippocampus. Overall, these data suggest that the role of mH2A in transcription in the mouse hippocampus is similar to its repressive role in other cell types^30,31^ and that this repressive function may be critical for its role in memory.

### mH2A1, but not mH2A2, is dynamically regulated during learning

**Fig. 5:**
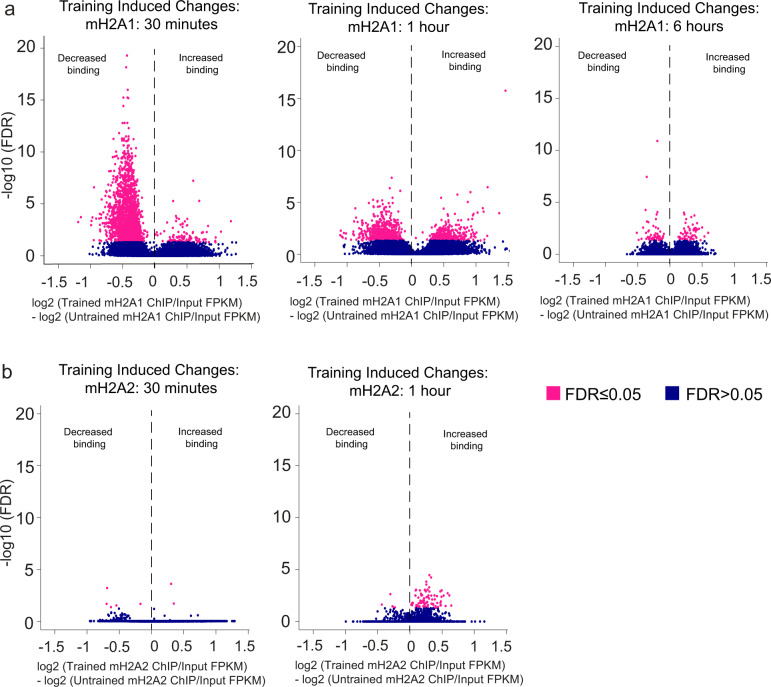
mH2A1, but not mH2A2 binding at broad domains is dynamically regulated during learning. **a** Differentially enriched regions (FDR ≤ 0.05) in untrained compared to trained mice were identified using DiffBind analysis of mH2A1 consensus peaks (using epic2 peak caller) 30 min, 1 h and 6 h after fear conditioning. **b** Differentially enriched regions (FDR ≤ 0.05) in untrained compared to trained mice were identified using DiffBind analysis of mH2A2 consensus peaks (using epic2 peak caller) 30 min or 1 h after fear conditioning. Data are plotted as ChIP FPKM divided by Input FPKM.

Histone variants H2A.Z and H3.3 are dynamically regulated during learning and in response to neuronal activity^4,5,7^, suggesting that learning-induced changes in histone variant binding are important for memory formation. To assess if mH2A1 and mH2A2 are also dynamically regulated during learning, we examined their occupancy in chromatin 30 min after fear conditioning, a time point when H2A.Z is subject to extensive regulation^4^. Learning-induced changes in histone binding were analysed using DiffBind (version 2.10.0) to compare consensus peaks in untrained vs. trained mice. Analysis of broad domains (from epic2) identified 2442 (i.e. 3% of all mH2A1 broad domains) regions with differential mH2A1 binding in fear conditioned compared to untrained mice (FDR ≤ 0.05), whereby 2316 (95%) loci had less mH2A1 signal and only 126 regions had more mH2A1 signal after fear conditioning (Fig. 5a, Supplementary Fig. 5 and Supplementary Data 9), suggesting that the majority of changes induced by training on the fear conditioning task involve the loss of mH2A1. Similarly, 2302 narrow mH2A1 peaks (MACS2) had reduced signal after training (i.e. 0.82% of all mH2A1 peaks; FDR ≤ 0.05) and only 127 peaks had increased signal after training (Supplementary Fig. 6), indicating that both broad domains and narrow peaks show rapid loss of mH2A1 binding 30 min after fear conditioning.

We previously showed that H2A.Z binding in the mouse hippocampus is highly dynamic 30 min after learning^4^ and returns to baseline within 2 h^5^, whereas changes in histone PTMs are typically observed 1 h after fear conditioning^32^. Moreover, epigenetic and transcriptional manipulations that influence memory if administered immediately before or after training have no effect if given 6 h after training^33^, indicating that the hippocampal consolidation window is closed at this time point. On this basis, we investigated mH2A1 binding 1 h and 6 h after training to represent an additional time point of memory consolidation, and the closure of the consolidation window, respectively^34^. Changes at epic2-identified mH2A1 broad peaks declined from 2442 at 30 min to 932 (60% had reduced signal with training) differentially bound regions 1 h after training and reverted to baseline levels by 6 h, when only 115 differentially bound peaks were identified, with 50% showing reduced signal in trained mice compared to naïve controls (Fig. 5a). These data suggest that mH2A1 responds rapidly to a learning event and reverts to baseline as the hippocampal memory consolidation window closes, similar to our previous report on H2A.Z^34^. Additionally, the mH2A binding pattern observed here is indicative of activity-mediated histone turnover, in which histones are transiently removed to promote transcriptional activity during learning and the same histone type is subsequently re-deposited into the nucleosome^7^. The fact that histone dynamics were restricted to the memory consolidation window suggests that mH2A1 may be especially important for regulating memory during this early consolidation phase.

In contrast, analysis of mH2A2 binding revealed a much less dynamic profile in response to learning. Specifically, only 7 differentially bound mH2A2 domains (based on epic2-identified broad peaks) were observed 30 min after fear conditioning (5 of 7 had significantly reduced binding) and 90 differentially bound domains were found 1 h after training (Fig. 5b). This pattern was also observed for MACS2 narrow peaks, with 54 peaks displaying a significantly reduced signal 30 min after fear conditioning and only 11 peaks 1 h after fear conditioning (Supplementary Fig. 6), suggesting a less dynamic role for mH2A2 compared to mH2A1 in response to fear conditioning. This difference in dynamics is consistent with evidence that mH2A2 is the predominant barrier in somatic cell reprogramming^17^, and hence it might be more resistant to losing its deposition in response to signalling events. Prior evidence shows that the histone variants H2A.Z and H3.3 that affect memory formation both exhibit activity-mediated turnover^4,5,7,35^, suggesting that the lack of mH2A2 dynamics in the present study may be associated with its weaker role in memory than the dynamically regulated histone mH2A1.

### Learning-induced mH2A1 removal occurs on upregulated genes

**Fig. 6:**
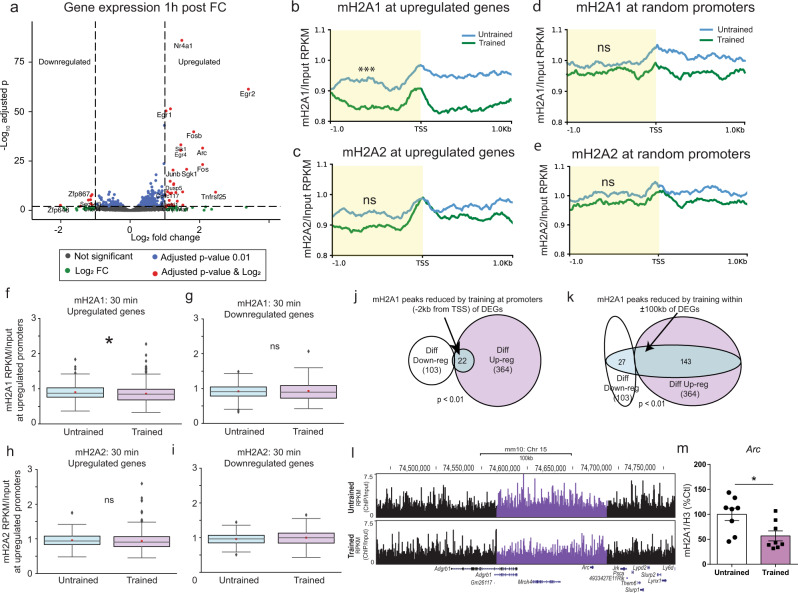
Learning-induced mH2A1 removal after 30 min occurs on upregulated genes. **a** Analysis of gene expression 1 h after contextual fear conditioning (*n* = 3 untrained, 2 trained). Blue and red circles indicate significantly altered genes. 364 of 467 differentially expressed genes (DEGs) were upregulated with fear conditioning. Average profile plot of **b** mH2A1 and **c** mH2A2 binding at TSS-flaking regions of upregulated genes. Upon training, mH2A1 signal is significantly reduced on promoters compared to untrained controls. There is no difference in mH2A2 binding on promoters of upregulated genes between trained and untrained mice. Average profile of **d** mH2A1 and **e** mH2A2 binding at promoters of randomly selected genes. There is no statistical difference in mH2A1 or mH2A2 binding on randomly selected promoters between trained and untrained mice. **f**, **g** Box plots comparing mH2A1 binding RPKM (mean across replicates) normalised to mean input RPKM values at promoters (−2 kb) in trained vs untrained mice on up- and down-regulated genes. Similarly, box plots were generated at **h** Upregulated DEGs and **i** Downregulated DEGs for comparison of mH2A2 binding between untrained and trained mice. Only mH2A1 shows a significant decrease in binding in trained compared to untrained samples for upregulated DEGs promoters, while mH2A2 show no significant changes in its binding in trained compared to untrained samples for either up or down regulated DEGs. **j** Venn diagrams demonstrating significant over-representation (*p* < 0.01, hyper-geometric test) of reduced mH2A1 signal on promoters (proximal regions) of differentially upregulated compared to promoters of upregulated DEGs. **k** Venn diagrams demonstrating significant over-representation (*p* < 0.01, hyper-geometric test) of reduced mH2A1 signal on extended regions (flanking ±100 kb from the centre of the peak) of upregulated compared to downregulated DEGs, implicating distal regulatory elements (e.g., enhancers/silencers). **l** University of California at Santa Cruz (UCSC) Genome Browser track (mm10 assembly) demonstrating significant decrease in mH2A1 binding 30 min after fear conditioning. Reads are normalised by dividing ChIP by Input. **m** To validate the learning-induced mH2A1 reduction observed with ChIP-seq, we fear conditioned a separate set of mice (*n* = 8/group) and measured mH2A1 binding (normalised to H3) 30 min later. As with ChIP-seq, there was a significant reduction in mH2A1 binding at the *Arc* promoter upon learning. Data are shown as mean ± SEM. (ns *p* > 0.05, *p < 0.05).

To assess if learning-induced changes in mH2A1 binding are associated with learning-induced changes in gene expression, we compared changes in ChIP-seq signal with changes in RNA-seq signal. RNA-seq was conducted 1 h after fear conditioning based on our previous observation that changes in H2A.Z binding 30 min after training better predict gene expression changes after 1 h rather than after 30 min^4^. First, we analysed gene expression changes that occur with contextual fear conditioning. Consistent with previous findings, DESeq2 analysis of RNA-seq read counts revealed that 78% (364 genes out of 467, under p adj < 0.01) of differentially expressed genes (DEG) 1 h after fear conditioning were upregulated (Fig. 6 and Supplementary Data 10), including the immediate early genes *Fos*, *FosB* and *Arc*, which have well-known roles in memory^36^.

As most DEGs (78%) were upregulated after training, we examined if fear conditioning is associated with altered mH2A1 and mH2A2 binding on upregulated genes. We compiled an average profile plot of mH2A1 and mH2A2 binding around the TSS of significantly upregulated (364) genes in trained and untrained mice. Fear conditioning was associated with significant reduction of mH2A1 binding at promoters (1 kb upstream of TSS) of upregulated genes compared to untrained mice (Fig. 6b, f based on quantitated input-normalised reads/million, *p* < 0.001, paired samples *t*-test), whereas mH2A2 deposition at the same region did not differ with training (Fig. 6c, h, *p* > 0.05, paired samples *t*-test). When similar analyses were conducted using a random set of promoters instead of promoters of upregulated genes, there were no differences in mH2A1 or mH2A2 binding in trained compared to untrained mice (Fig. 6d, e; *p* > 0.05, paired samples *t*-test), suggesting that loss of mH2A1 signal with training preferentially occurs on promoters of upregulated DEGs. We also found no difference in mH2A1 binding between untrained and trained mice on promoters of downregulated DEGs for mH2A1 and mH2A2 (Fig. 6g, i and Supplementary Data 5).

To assess mH2A1 binding on learning-regulated genes across time, we compared mH2A1 binding on upregulated *vs* downregulated genes separately for untrained (Supplementary Fig. 7a, c, e) and trained mice at each of the 3 time points (30 min, 1 h, 6 h). mH2A1 binding in trained mice was significantly lower on upregulated compared to downregulated DEGs (*p* < 0.01, unpaired samples *t*-test, Supplementary Fig. 7b) 30 min after training, but not at other time points (1 h, 6 h) (Supplementary Fig. 7d, f). In contrast, mH2A1 binding did not differ on upregulated compared to downregulated DEGs in untrained mice at any time point (30 min, 1 h and 6 h). Lastly, mH2A1 binding 6 h after fear conditioning displayed no significant changes between trained and untrained samples at either upregulated or downregulated DEGs at 1 h, suggesting that epigenomic binding of mH2A1 at promoters returns to baseline levels as the hippocampal memory consolidation window closes (*p* > 0.05, paired samples *t*-test, Supplementary Fig. 7g, h).

In a reverse approach, we used regions with differential mH2A1 loss upon fear-conditioning (identified by DiffBind using epic2 peaks) as bait to test their association with gene expression. If learning-induced loss of mH2A1 binding occurs on upregulated genes, we reasoned that mH2A1 removal may preferentially occur on the regulatory regions of upregulated compared to downregulated DEGs. To test this hypothesis, we looked at the overlap between the promoters (within 2 kb of the TSS) of all DEGs and the regions that had a significant loss of mH2A1 during learning. Of 23 DEG promoters that lost mH2A1, 22 (95.7%) were on significantly up-regulated genes, such that only one site of lost mH2A1 binding overlapped with a downregulated gene, resulting in a significant overrepresentation of mH2A1 loss on the promoters of upregulated genes (Hyper-geometric test: *p* < 0.01) (Fig. 6j). A relationship between gene expression and mH2A binding was not found for narrow peaks (MACS2), indicating that broad domains lose mH2A1 more than narrow peaks on upregulated genes. This observation is consistent with a recent report that changes in broad domains of H3K4me3 have a stronger association with learning-induced gene expression compared to changes in H3K4me3 peaks^37^.

Gene expression is also influenced by distal regulatory elements, such as enhancers and silencers, leading us to incorporate these elements into a similar over-representation analysis. Upregulated DEG promoters were over-represented within the ±100 kb of regions with reduced mH2A1 signal were (143 out of 170 genes; Hyper-geometric test: *p* < 0.01). This analysis captured 143 out of the total 346 up-regulated genes, which is a significant enrichment (Hyper-geometric test: *p* < 0.0001) considering the total number of genes in the ±100 kb range (Fig. 6k), suggesting that learning-induced removal of mH2A1 occurs on promoters and distal regions within 100 kb of upregulated genes. In contrast, regions that lost mH2A1 binding 1 h after fear conditioning did not overlap with upregulated genes at 1 h (Supplementary Fig. 8). As an additional validation of reduced mH2A1 binding with learning, we also observed reduced mH2A1 signal at the *Arc* gene (Fig. 6l) in a separate group of fear conditioned mice using ChIP-qPCR. This approached confirmed reduced mH2A1 binding on the *Arc* promoter 30 min after fear conditioning (*F*_1,14_ = 7.23, *p* = 0.018), thus validating learning-induced removal of mH2A1 binding observed with ChIP sequencing (Fig. 6m and Supplementary Data 6).

### Link between transcription and macroH2A in cultured neurons

Fear conditioning data suggest that mH2A1 depletion regulates transcription in the brain and that changes in mH2A1 binding co-occur with changes in transcription. To test if this relationship reflects a general response to neuronal activity, we utilised a primary neuronal culture model, in which neurons are depolarised with potassium chloride (KCl) to induce neuronal activation. Gene expression was measured in control (infected with scrambled shRNA) or mH2A1-deficient (infected with AAV vector carrying shRNA against mH2A1) neurons 15 min or 1 h after KCl washout. *Fos* expression increased 15 min and 1 h after KCl in scramble control neurons, but did not increase until 1 h in mH2A1-deficient neurons. Moreover, *Fos* levels were higher in mH2A1-deficient compared to scramble control neurons at all time points (Main effect of KCl: *F*_1,30_ = 37.78, *p* < 0.001; main effect of Virus: *F*_1,30_ = 14.31, *p* < 0.01; interaction: *F*_2,30_ = 2.76,*p* = 0.079). *Arc* expression was induced to a similar extent in mH2A1 deficient and control neurons 1 h after KCl, with a trend toward higher *Arc* levels in mH2A1-depleted neurons 1 h after KCl (*p* = 0.06) (main effect of Virus: *F*_1,29_ = 3.82, *p* = 0.06; main effect of KCl: *F*_2,29_ = 78.80, *p* < 0.001; interaction *F*_2,29_ = 4.7, *p* = 0.02). mH2A1 depletion resulted in higher basal levels of *Gadd45b* (main effect of Virus: *F*_1,30_ = 74.63, *p* < 0.001), and KCl induced increased expression 1 h after KCl only in scrambled control neurons (main effect of KCl in shScr: *F*_2,18_ = 12.73, *p* < 0.001; ns in sh*H2afy*: *F*_2,15_ = 2.50, *p* = 0.12), indicating that both neuronal activity and mH2A1 regulate gene expression in neurons (Supplementary Fig. 9).

To determine if activity-induced transcription co-occurs with mH2A1 dynamics, we assessed mH2A1 binding using the same protocol as for gene expression, but in uninfected neurons. As with fear conditioning, mH2A1 was rapidly removed in response to neuronal activity, whereby mH2A1 binding decreased after 15 min for all genes examined. By 1 h, mH2A1 binding either returned to baseline (*Fos*: *F*_2,14_ = 5.02, *p* = 0.02; *Gadd45b*: *F*_2,14_ = 4.78, *p* = 0.03) or increased (for *Arc* only: *F*_2,15_ = 12.11, *p* < 0.001) compared to unstimulated neurons, indicating a similar pattern of mH2A1 removal and re-incorporation as in the brain. Thus, studies in a cultured neuronal system support the observation that mH2A1 is transiently removed from upregulated genes and that mH2A1 depletion impacts gene activity (Supplementary Fig. 9)

## Discussion

Here, we report that the histone variant mH2A is an isoform-specific regulator of memory formation, whereby mH2A1 has a more pronounced effect on memory than mH2A2. The stronger effect of mH2A1 than mH2A2 on memory was associated with stronger repressive effects on transcription, and with preferential reduction in mH2A1 over mH2A2 binding in response to learning. Notably, mH2A1 depletion drastically impaired learning-induced gene expression, suggesting that mH2A1 is required for effective transcriptional induction during learning.

Impaired learning-induced transcription in mH2A1-depleted mice is particularly interesting because of its primary role as a transcriptional repressor, as evident by extensive upregulation of basal transcription when mH2A1 is depleted (Fig. 2b). However, our ChIP sequencing data show that mH2A1 is also bound to inducible genes, as evidenced by changes in mH2A1 occupancy on upregulated genes after learning. Indeed, despite its primarily repressive role, mH2A1 is found on both repressed and inducible genes^38^ and its binding is required for the induction of a subset of genes in non-neuronal systems^39,40^, indicating that mH2A1 may facilitate binding of transcriptional activators that promote learning. Alternatively, mH2A1 is also a powerful inhibitor of spurious transcription^38^ and as such, transcriptional de-repression in mH2A1-deficient mice under baseline conditions can be interpreted as transcriptional noise that disrupts a normal transcriptional response to a learning event. Accordingly, studies of other repressive marks, including DNA methylation, show that loss of repressive marks increases basal gene expression and impairs memory^41–44^, suggesting that repressive mechanisms may facilitate a transcriptional response to learning by silencing spurious transcription.

Several studies have demonstrated that histones H2A.Z and H3.3 play a role in memory and are dynamically regulated by neural activity and learning^4,5,7,35^, suggesting that histone dynamics may be critical for memory. Data from the present study extend these findings to mH2A by showing that mH2A1 is more dynamic of the two isoforms and has a stronger role in memory than mH2A2. However, understanding the relevance of chromatin dynamics in memory is ongoing, as learning induces extensive changes in histone variants, histone post-translational modifications, DNA methylation and chromatin accessibilit^4,32,45,46^, but a comparatively small association exists between these changes and learning-induced gene expression. Even when we do find a link between learning-induced changes in histone dynamics and gene expression, as we do here, it is not clear if loss of mH2A1 is causal for learning-induced gene upregulation. Nevertheless, both H2A.Z^4^ and mH2A1 are dynamically regulated during learning, suggesting that stimulus-induced histone variant dynamics may have an important role in memory.

The unique effects of mH2A1 and mH2A2 on memory and transcription are especially interesting because these histones exhibit similar binding patterns and a similar relationship with basal gene expression. Thus, despite their general similarity in function, they differ in their relative stability in chromatin. These findings complement studies of cellular reprogramming, which report largely overlapping roles for mH2A1 and mH2A2, but nevertheless show that mH2A2 is a more effective barrier to cellular reprogramming than mH2A1, indicating potential resistance to removal by reprogramming stimuli^17^. A preferential role of mH2A1 in memory and gene regulation may be particularly relevant in the brain, given that mH2A1 is a biomarker for Huntington’s disease^11^ and its dysregulation may also be involved in neurodegeneration.

Despite its primarily repressive effect on transcription, mH2A1 depletion reduced the expression of genes critical for normal synaptic function, indicating that mH2A1 may also influence memory by regulating dendritic morphology and synaptic function. Overall, we suggest that mH2A1 regulates memory by maintaining inducible genes in a repressed state until an appropriate stimulus leads to its removal and de-repression of genes involved in synaptic plasticity.

The positive effect of mH2A on memory contrasts the negative effect of the histone variant H2A.Z, whereby H2A.Z depletion in area CA1 enhances memory^3–5^. Indeed, H2A.Z and mH2A differ on several key parameters. Whereas mH2A predominantly binds to genes with low basal expression, H2A.Z binding is highest on highly expressed genes in the mouse hippocampus^4^. Consistently with their opposing effects on basal transcription, these histones also demonstrate opposing interactions with methylated DNA in non-neuronal cells, whereby mH2A tends to co-localise with methylated DNA^47^ and H2A.Z is depleted from methylated regions^48^. Similarly, mH2A levels tend to increase upon cellular differentiation whereas H2A.Z levels simultaneously decrease^17^, reinforcing opposite roles for the two histones.

Although the relationships between H2A.Z, mH2A1 and DNA methylation have not been tested in the brain, our evidence for opposing effects of mH2A and H2A.Z on memory implicate these histones as key interacting elements in epigenetic regulation of memory. This is especially interesting because both histones are variants of H2A and as such, they have the capacity to replace one another in chromatin. Although such replacement has not been directly tested in any tissue, some evidence in non-neuronal cells suggests that H2A.Z and mH2A may replace one another when previously unmethylated DNA becomes methylated^49^. In addition, the macro domain of mH2A associates with histone deacetylases and as such, has been linked with deacetylation of neighbouring histones^50^, suggesting that histone variants contribute to shaping the epigenetic landscape of well-established regulators of memory, such as DNA methylation and histone acetylation^34^.

Overall, data from the present study and from our work on H2A.Z indicate that H2A variants are a functionally diverse family of histones that have unique effects on memory. Their function is further diversified through distinct genes encoding each histone type, which may allow for the maintenance of stable chromatin states while also allowing for dynamic chromatin regulation in response to experience. Understanding how individual histone types regulate memory is a vital first step in identifying the role of their interactions in memory formation and neural plasticity. The opposing effects of histone H2A.Z and mH2A on memory provide insights into the functional diversity of histone variants, with implications for combinatorial effects of H2A variants on neural plasticity and behavioural regulation.

## Methods

### Animals

Male C57BL/6 J mice (Jackson Laboratories) of ~9–12 weeks of age were used for the experiments. Mice were pair housed upon arrival and food and water were available ad libitum. Mice were given at least one week to habituate to the colony before cage mates were randomly assigned to the behavioural treatment group, such that mice in the same cage always belonged to the same test group. All procedures were approved by the University of Toronto Animal Care Committee and performed in accordance with the Canadian Council on Animal Care guidelines.

### Protein extraction and western blotting

A fluorescent lamp was used to dissect the area of the hippocampus expressing eGFP in mice who received viral injections. Tissues were homogenised using a dounce homogenizer in RIPA buffer (50 mM Tris HCl pH 7.4, 150 mM NaCl, 10% NP-40, 0.5% sodium deoxycolate, 0.1% SDS) supplemented with Protease Inhibitor Cocktail (Cell Signalling). Homogenates were incubated for 20 min on ice, centrifuged at maximum speed at 4 °C for 15 min, and the supernatant was collected. Proteins were separated on a 15% SDS-PAGE and transferred to a PVDF membrane. Membranes were blocked in PBS-T containing 5% milk and incubated with primary antibodies against mH2A1(1:500, Abcam Cat#37264), mH2A2 (1:500, Active Motif Cat#39873) and H3 (1:10,000, Cell Signaling Cat#2650) overnight at 4 °C. After washing, membranes were incubated with the appropriate secondary antibody (1:10,000 Life Technologies) for 1 h at RT. Detection was performed by chemiluminescence using Luminata Forte HRP substrate (Millipore, Cat#WBLUF0500) and visualised with Luminescent image analyser (ImageQuant LAS 500).

### AAV production

Viral vector-mediated knockdown of mH2A1 and mH2A2 was achieved using a bicistronic vector encoding *H2afy* (encodes mH2A1) or *H2afy2* (encodes mH2A2) short hairpin RNA (shRNA) driven by the U6 promoter and CMV driving GFP expression and packaged in AAV serotype DJ (sh*H2afy*: 4.42 × 10^12^ genome copies per ml; sh*H2afy2*: 9.00 × 10^12^) at St. Jude Children’s Research Hospital. Briefly, AAV vector production was initiated by PEI- (polyethyleneimine “max”, Polysciences cat no 24765) mediated plasmid transfection. After three days, cell pellets were harvested and lysed by repeated freeze-thawing, while supernatants were pegylated with 40% polyethylene glycol. Both were treated with benzonase and then subjected to caesium chloride step gradient separation. The lower, virus-containing fraction was collected and dialysed against PBS and concentrated by Amicon filter (100 kDa) before titering by qPCR. AAV vectors were titered by qPCR using serial dilutions of virus compared to linearised plasmid standards (25 μl reactions with primers to CMV promoter: Forward CMV primer: ATATGCCAAGTACGCCCCCTATTGAC and Reverse CMV primer: ACTGCCAAGTAGGAAAGTCCCATAAGGTC, performed on Applied Biosystems 7500 machine). shRNA sequence for *H2afy* was GTTTGTGATCCACTGTAATAG and the shRNA sequence used for *H2afy2* was GCTACTGAAAGGAGTGACTAT.

### Stereotaxic surgery for mH2A depletion

Mice were anaesthetized with isoflurane and secured to a stereotaxic apparatus. Viral particles were bilaterally delivered into the dorsal CA1 (anterior/posterior (AP) − 1; medial/lateral (ML) ± 1.5; dorsal/ventral (DV) − 1.6; 1 μl per hemisphere) at a rate of 225 nl min^−1^, with 2 weeks allowed for recovery before behavioural testing.

### Fear conditioning

Mice were handled for 3 days before contextual fear conditioning, which consisted of 2 min of exploration, followed by 3 electric foot-shocks (0.5 mA, 2 s) administered 1 min apart, with an additional 1 min of exploration before removal from the chamber. Untrained control mice remained in their home-cage. Memory recall was assessed 24 h after training and consisted of re-exposure to the context for 3 min in the absence of shock. Percent of time spent freezing was taken as an index of memory and was scored by automated software (FreezeFrame, Coulbourn Instruments). For ChIP sequencing experiments, tissue was collected 30 min after training and for RNA sequencing, tissue was collected 1 h after training based on results of previous studies with H2A.Z^4^.

### Open field

Mice were transported to the testing room in their home cages (covered) for 30 min of habituation on the day of testing. Mice were placed into the testing chamber (45 cm × 45 cm × 45 cm) for 6 min and tested in pairs. Video was recorded using a camera (Microsoft LifeCam Studio) and scored with EthoVision XT 8.5. Total time spent in the chamber was scored using EthoVision XT 8.5 (Noldus Information Technology, Wageningen, The Netherlands).

### Object in Place

Mice underwent three 5 min sample phases with 5 min in between each sample. In all sample phases, mice were presented with the same 4 distinct objects, with 1 object placed in each corner of the OF, away from the walls. Following the sample phase, there was a 24 h retention delay to assess long-term memory. After the retention delay, mice underwent a 5 min choice phase where they viewed the same four objects, but two objects had switched locations (right or left, counterbalanced across mice), thus creating a novel side. Memory is inferred from the preferential exploration of objects on the novel side, as captured by the Discrimination Ratio (DR):

1 $${{{\mathrm{DR}}}} 	= [({{{\mathrm{novel}}}}\; {{{\mathrm{object}}}}\; {{{\mathrm{exploration}}}} - {{{\mathrm{familiar}}}}\; {{{\mathrm{object}}}}\; {{{\mathrm{exploration}}}})\\ 	\quad/({{{\mathrm{novel}}}} + {{{\mathrm{familiar}}}} \;{{{\mathrm{object}}}}\; {{{\mathrm{exploration}}}})].$$

### Chromatin immunoprecipitation for sequencing

Whole CA1 samples were crosslinked with 1% formaldehyde for 10 min at 37 °C, quenched with 1.25 M glycine, then washed with PBS containing protease inhibitors (Cell Signaling Cat# 5872). Tissue was homogenised in 500 μl of buffer A (0.25 M sucrose, 60 mM KCl, 15 mM NaCl, 10 mM Pipes pH 6.8, 5 mM MgCl_2_. 0.5% triton) then pre-sonicated in 500 μl of buffer B (50 mM NaCl 10 mM Pipes pH 6.8, 5 mM MgCl_2_) at 35% amplitude (3 times for 10 s, 30 s rest between sonications) before digestion with micrococcal nuclease (Cell Signaling Cat#10011) for 20 min at 37 °C. Lysate was centrifuged at 17 000 RCF for 5 min at 4 °C, aliquoted and diluted 1:8 with ChIP dilution buffer (16 mM Tris pH 7.3, 0.01% SDS, 1% Triton X-100, 1.2 mM EDTA, 160 mM NaCl). Aliquots were treated with protein G magnetic beads (Millipore, Cat#16-662) that were pre-washed in 2% BSA for 2 h and 5 μg of either mH2A1 (Abcam cat# 37264 for 30 min; Thermo Fisher cat# Ma5-24696 for 1 h and 6 h time points due to prolonged supply issue from Abcam) or 15 μg of mH2A2 (Abcam cat#4173) overnight at 4 °C with rotation. After overnight incubation in primary antibody, beads were pelleted with a magnetic separator and washed sequentially with low-salt (20 mM Tris 7.3, 0.1% SDS, 1% Triton X-100, 2 mM EDTA and 150 mM NaCl), high-salt (20 mM Tris 7.3 pH, 0.1% SDS, 1% Triton X-100 and 495 mM NaCl), LiCl immune complex (EMD Millipore) and TE buffers (10 mM Tris pH 7.3, 1 mM EDTA). Immune complexes were extracted using TE buffer with proteinase K (Cell Signalling Cat#5872), heated at 65 °C for 2 h, followed by 95 °C for 10 min. Samples were purified with the Qiagen PCR cleanup kit and sent for next-generation sequencing at Genome Quebec.

### Antibody validation

Although there are many commercially available ChIP-validated antibodies against mH2A1, we performed an in-lab validation of a commercially-available mH2A2 antibody from Abcam for ChIP (Supplementary Fig. 10). First, we showed that both Abcam and Active Motif (Cat#39873) mH2A2 antibodies produce enrichment above IgG on promoters of *Arc* (*F*_2,9_ = 14.59, *p* = 0.005, all post hoc < 0.05), *Gria4* (F2,9 = 7.60, *p* = 0.2, all post hoc < 0.05) and *Fos* (*F*_2,9_ = 6.09, *p* = 0.04, all post hoc *p* < 0.05), with Abcam showing stronger enrichment on *Fabp1* (*F*_2,6_ = 12.79, *p* = 0.007, Abcam vs IgG *p* = 0.026; Active Motif vs IgG *p* = 0.08) compared to Active Motif in tissue from the mouse hippocampus. We further validated the specificity of the Abcam antibody for mH2A2 over mH2A1 by infecting neurons with shRNA against each gene, followed by ChIP. In each case, the mH2A2 ChIP signal was reduced with mH2A2 depletion and was not affected by mH2A1 depletion, indicating the suitability of this antibody for ChIP-seq experiments (*Arc*: *F*_2,11_ = 8.23, *p* = 0.007; Gria4: *F*_2,11_ = 6.81, *p* = 0.01; *F*_2,11_ = 10.40, *p* = 0.03; *Fabp1*: *F*_2,11_ = 5.34, *p* = 0.02; For all 4 genes, post hoc comparisons for *H2afy2* vs sh*Scr* and sh*H2afy2* vs sh*H2afy* had *p* < 0.05).

Due to unexpected antibody supply issues with Abcam that were out of our control, mH2A1 ChIP antibody we used at 30 min was not available when we conducted experiments on 1 h and 6 h time points. To ensure that the two antibodies (anti-mH2A1 from Abcam and Thermo Fisher) performed similarly, we examined overlap between samples that received the same antibody, as well as the overlap between samples that received different antibodies.

Samples collected at 30 min with the Abcam antibody had 73% mH2A1 peaks overlapping amongst themselves and the samples collected at the 1 h time point with the Thermo Fisher antibody had 72% overlap amongst themselves. The overlap between the peaks of two mH2A1 antibodies (and hence, the two time points) was 51%. This overlap is consistent with a 59% overlap in common peaks for all samples at 30 min compared to 1 hr for mH2A2 signal, which utilised the same antibody for both time points. Thus, despite using two different antibodies at two separate sequencing runs, we detected 30,273 common peaks across all naïve samples for mH2A1, while 27,542 shared peaks for mH2A2 (same antibody both times), indicating that the two mH2A1 antibodies picked up similar number of consensus signals at the two time points as done by same antibody for mH2A2. The overlap between two separate mH2A1 antibodies also provides further validation of the specificity of our sequencing signal for mH2A1.

### Bioinformatics

#### ChIP-seq

DNA libraries were sequenced (~50 million total 100 base-pair paired end reads) on an Illumina sequencing platform (HiSeq 4000). Read quality was assessed using FastQC (v.0.11.2) and adaptors were trimmed using Trim Galore (v.0.4.5) running Cutadapt (v2.6) and only paired reads were retained in the analysis. Reads were aligned to the UCSC Mus Musculus mm10 reference genome using Bowtie2 (v2.3.4.2)^51^. multiBamSummary and plotCorrelation from deepTools^52^ was used for correlation analysis of mH2A1 and mH2A2 binding. Heatmap and average profile plots giving mean read counts per million mapped reads for all specified regions at TSSs were generated using ngs.plot^53^. bamCompare, bigwigCompare and plotProfile from deepTools were used for generating the input normalised mH2A ChIP RPKM average profile plot to compare the trained and untrained condition using two mice under each condition. For ChIP signal visualisation at University of California at Santa Cruz (UCSC) Genome Browser, the bamCompare from deepTools was used to generate bedgraphs with input normalisation using ratio operation. Broad and diffusely enriched domains (regions enriched for reads compared to input) were identified using epic2 (v 0.0.39; FDR < 0.05) and MACS2 (v 2.2.7.1; *p* value cutoff = 5e-3; broad-cutoff as 0.05 or 0.01) and consensus regions for all samples were assembled based on enrichment in at least two samples. Differential analyses for mH2A1 and mH2A2 binding in Untrained and Trained conditions were carried out using DiffBind (v.2.10.0)^54^ with R (v.3.5.0) and significance was set at FDR < 0.05. Enriched regions from epic2 and MACS2 for each sample along with respective bam files for ChIP experiment and input control under respective training conditions were used as input for DiffBind which uses the DESeq2 package for differential analysis. For all peaks analysed by DiffBind, raw read counts were identified for both ChIP and input control using featureCounts (v.1.6.4) to calculate the ‘ChIP divided by input’ normalised FPKM fold changes upon training. For the statistical tests of mH2A binding at promoters of DEGs, the mean of RPKM values for all the samples under respective trained or untrained condition were normalised using the respective mean of input control reads.

#### RNA-Seq

RNA was extracted using RNeasy Mini Kit (Qiagen). Samples were sent for next generation sequencing at Genome Quebec (Fig. 2b, c) or Princess Margaret Genomics Centre (Fig. 2e, f) using Illumina HiSeq4000 (~100 million total 100 base-pair paired end reads). Raw paired-end reads were assessed for quality using FastQC (v.0.11.2) and adaptors were trimmed using Trim Galore (v.0.4.5) running Cutadapt (v.2.6) or BBDuk. The reads were aligned to the Mus Musculus mm10 reference genome using STAR (*v* ≥ 2.6.0c)^55^. Read distribution was assessed using RseQC package (v2.3.7). featureCounts (Subread package *v* ≥ 1.26.1) was used to get the read counts. As ribo-depletion kit was used for RNA-seq for Fig. 2e, f, we used the whole transcript length from transcription start to end for read counting using featureCounts. Two-condition differential expression for specific comparisons was assessed using DEseq2 (v 1.22.2)^56^ in R using 0.01 adjusted p-value cut-off. EnhancedVolcano package was used to generate the volcano plots. Using the average read counts across the biological replicates under different conditions, gene with non-zero average read counts were split into three categories (High, Medium and Low) corresponding to top 33.3%, middle 33.3% and bottom 33.3% expressed genes.

### Primary cortical neurons and treatments

Cortices were isolated from E16.5 pups, washed twice with HBSS (GIBCO, Cat# 14175095), digested with 0.25% Trypsin (Life Technologies, Cat# 15050065) and mechanically dissociated by pipetting. Cells were plated on poly-L-lysine (Sigma, Cat# P2636; 0.1 mg/mL) at a density of 500000 cells/well on 6-multiwell plates for both qPCR and ChIP experiments. Neurons were grown in Neurobasal medium (GIBCO, Cat# 21103049) supplemented with L-glutamine (GIBCO, Cat# 25030081) and B-27 (Life Technologies, Cat# 17504044). For gene expression analyses, neurons were infected at 7 days in vitro (DIV) using 5000 vg/cell. 7 days after infection, KCl was added to a final concentration of 55 mM directly to the culture media for 30 min at 37 °C. After, KCl-containing media was replaced with fresh media for 15 or 60 min at 37 °C. Vehicle groups were left untreated. For RNA expression analysis, RNA was extracted with EZ-10 total RNA extraction kit (Biobasic, Cat# BS82322) with an added DNase step (Qiagen, Cat# 79254). Complementary DNA was synthesised using high-capacity cDNA Reverse Transcription kit (Applied Biosystems, Cat# 4368814). Primers were designed in the lab to detect levels of the indicated transcripts and data were normalised to the geometric mean of β-actin and GAPDH.

For ChIP, neurons were fixed on plates in PBS 0.5% formaldehyde, quenched with 1.25 M glycine after 5 min at RT, then washed 6X with ice-cold PBS and scraped in 300uL ChIP lysis buffer (50 mM Tris pH 7.4, 10 mM EDTA, 1% SDS). ChIP lysates were sonicated using a Bioruptor® (30 cycles, 30 s on 30 s off; Diagenode) at 4 °C., centrifuged at 17000 g for 1 min at 4 °C and the supernatant was aliquoted and diluted with ChIP dilution buffer (16 mM Tris pH 7.3, 0.01% SDS, 1% Triton X-100, 1.2 mM EDTA, 160 nM NaCl). Aliquots were incubated with 20 μL of Protein G magnetic beads (Milipore, Cat# 16-662) and 1uL of mH2A1 (Thermo Fisher, Cat# MA5-24696) or H3 (Cell Signaling, Cat# 2650 S) antibody overnight at 4 °C. The next day, immunoprecipitates were washed sequentially with low-salt (20 mM Tris pH 7.4, 0.1% SDS, 1% Triton X-100, 2 mM EDTA, 150 mM NaCl), high-salt (20 mM Tris pH 7.4, 0.1% SDS, 1% Triton X-100, 2 mM EDTA, 500 mM NaCl), LiCl (Millipore, Cat# 20-156) and TE (10 mM Tris pH 7.4, 1 mM EDTA) buffers while rotating for 5 min at RT at each wash step. Immune complexes and inputs were resuspended in TE buffer and proteinase K (Roche, Cat# 3115828001) and heated at 65 °C for 2 hr followed by 95 °C for 10 min. Samples were cooled to RT and genomic DNA was purified with a PCR Purification Kit (Biobasic, Cat# BS664). Primers were designed in the lab to detect specific genomic sequences. ChIP data were calculated as %input and normalised to respective control samples.

### Statistics and reproducibility

For behavioural testing and cell culture experiments, analyses were conducted using SPSS 25 (IBM). Data were analysed using one-way ANOVA with Virus as the independent variable (scramble, sh*H2afy*, sh*H2afy2*). When only 2 groups were compared, independent samples t test was used. Significance was set at *p* < 0.05, two tailed. Sample sizes for specific experiments are as follows. In Fig. 1a, we used *n* = 7 mice in scramble control group, *n* = 9 mice in *H2afy* depleted group and *n* = 6 mice in *H2afy2* depleted group. In Fig. 1b, c, we used *n* = 34 mice in scramble control group, *n* = 24 mice in *H2afy* depleted group and *n* = 18 in *H2afy2* depleted group. For Fig. 1d, we used *n* = 10 mice per group. For Fig. 1e, we used *n* = 16 scramble control mice, *n* = 17 *H2afy* depleted mice and *n* = 18 *H2afy2* depleted mice. For Fig. 6m, we used *n* = 8 mice/group. For RNA seq in Fig. 2b–e, we used 3 mice/group and for Fig. 6a, we used 3 untrained and 2 trained mice. For mH2A1 ChIP seq, we used 3 untrained mice and 2 trained mice at the 30 min time point, 2 mice/group at the 1 h time point and 5 mice/group at the 6 h time point. For mH2A2 ChIP seq, we used 3 untrained mice and 2 trained mice at the 30 min time point and 3 mice/group at the 1 h time point.

### Reporting summary

Further information on research design is available in the Nature Research Reporting Summary linked to this article.

## Supplementary information

Supplementary Information

Description of Additional Supplementary Files

Supplementary Data 1

Supplementary Data 2

Supplementary Data 3

Supplementary Data 4

Supplementary Data 5

Supplementary Data 6

Supplementary Data 7

Supplementary Data 8

Supplementary Data 9

Supplementary Data 10

Reporting Summary

## Data Availability

Source data for RNA and ChIP sequencing experiments are deposited on GEO under accession number GSE147445. Supplementary Data 1 contains the source values underlying Fig. 1a. Supplementary Data 2 contains the source values underlying Fig. 1b, c. Supplementary Data 3 contains the source values underlying Fig. 1d. Supplementary Data 4 contains the source values underlying Fig. 1e. Supplementary Data 5 contains the source values underlying Fig. 6f–i. Supplementary Data 6 contains the source values underlying Fig. 6m. Supplementary Data 7 contains RNA sequencing data relevant for Fig. 2b, c. Supplementary Data 8 contains RNA sequencing data relevant for Fig. 2e, f. Supplementary Data 9 contains ChIP sequencing data relevant for Fig. 5. Supplementary Data 10 contains RNA sequencing relevant for Fig. 6a. Supplemental Fig. 1 contains uncropped blots from Fig. 1a.

## References

[1] Campbell RR, Wood MA (2019). How the epigenome integrates information and reshapes the synapse. Nat. Rev. Neurosci..

[2] Lattal KM, Wood MA (2013). Epigenetics and persistent memory: implications for reconsolidation and silent extinction beyond the zero. Nat. Neurosci..

[3] Narkaj, K. et al. Blocking H2A.Z incorporation via tip60 inhibition promotes systems consolidation of fear memory in mice. *eNeuro* **5** (2018). 10.1523/ENEURO.0378-18.2018 PMC6223110 30417078

[4] Stefanelli G (2018). Learning and Age-Related Changes in Genome-wide H2A.Z Binding in the Mouse Hippocampus. Cell Rep..

[5] Zovkic IB, Paulukaitis BS, Day JJ, Etikala DM, Sweatt JD (2014). Histone H2A.Z subunit exchange controls consolidation of recent and remote memory. Nature.

[6] Lepack AE (2016). Aberrant H3.3 dynamics in NAc promote vulnerability to depressive-like behavior. Proc. Natl Acad. Sci. USA.

[7] Maze I (2015). Critical role of histone turnover in neuronal transcription and plasticity. Neuron.

[8] Dunn, C.J. et al. Histone hypervariants H2A.Z.1 and H2A.Z.2 play independent and context-specific roles in neuronal activity-induced transcription of Arc/Arg3.1 and other immediate early genes. *eNeuro* **4**, ENEURO.0040-17.2017 (2017). 10.1523/ENEURO.0040-17.2017 PMC5569379 28856239

[9] Millar CB (2013). Organizing the genome with H2A histone variants. Biochem J..

[10] Kamakaka RT, Biggins S (2005). Histone variants: deviants?. Genes Dev..

[11] Hu Y (2011). Transcriptional modulator H2A histone family, member Y (H2AFY) marks Huntington disease activity in man and mouse. Proc. Natl Acad. Sci. USA.

[12] Ramzan F (2020). Sex-specific effects of the histone variant H2A.Z on fear memory, stress-enhanced fear learning and hypersensitivity to pain. Sci. Rep..

[13] Ramzan, F., Baumbach, J., Monks, A.D. & Zovkic, I.B. Histone H2A.Z is required for androgen receptor-mediated effects on fear memory. *Neurobiol. Learn. Mem.* **175**, 107311 (2020). 10.1016/j.nlm.2020.107311 32916283

[14] Pehrson JR, Fried VA (1992). MacroH2A, a core histone containing a large nonhistone region. Science.

[15] Costanzi C, Pehrson JR (2001). MACROH2A2, a new member of the MARCOH2A core histone family. J. Biol. Chem..

[16] Gamble MJ, Kraus WL (2010). Multiple facets of the unique histone variant macroH2A: from genomics to cell biology. Cell Cycle.

[17] Gaspar-Maia A (2013). MacroH2A histone variants act as a barrier upon reprogramming towards pluripotency. Nat. Commun..

[18] Gonzalez-Munoz E, Arboleda-Estudillo Y, Chanumolu SK, Otu HH, Cibelli JB (2019). Zebrafish macroH2A variants have distinct embryo localization and function. Sci. Rep..

[19] Pehrson JR, Changolkar LN, Costanzi C, Leu NA (2014). Mice without macroH2A histone variants. Mol. Cell Biol..

[20] Barker GR, Warburton EC (2011). When is the hippocampus involved in recognition memory?. J. Neurosci..

[21] Chen EY (2013). Enrichr: interactive and collaborative HTML5 gene list enrichment analysis tool. BMC Bioinform..

[22] Kuleshov MV (2016). Enrichr: a comprehensive gene set enrichment analysis web server 2016 update. Nucleic Acids Res..

[23] Koh JY, Kim HN, Hwang JJ, Kim YH, Park SE (2019). Lysosomal dysfunction in proteinopathic neurodegenerative disorders: possible therapeutic roles of cAMP and zinc. Mol. Brain.

[24] Cortes CJ, La Spada AR (2014). The many faces of autophagy dysfunction in Huntington’s disease: from mechanism to therapy. Drug Discov. Today.

[25] Douet J (2017). MacroH2A histone variants maintain nuclear organization and heterochromatin architecture. J. Cell Sci..

[26] Buschbeck M, Di Croce L (2010). Approaching the molecular and physiological function of macroH2A variants. Epigenetics.

[27] Stovner EB, Saetrom P (2019). epic2 efficiently finds diffuse domains in ChIP-seq data. Bioinformatics.

[28] Bhowmik S, Gagnuly A (2019). A review article on ChIP-Seq tools: MACS2, HOMER, SICER, PEAKANNOTATOR and MEME. Int. J. Chem. Environ. Sci..

[29] Buschbeck M (2009). The histone variant macroH2A is an epigenetic regulator of key developmental genes. Nat. Struct. Mol. Biol..

[30] Doyen CM (2006). Mechanism of polymerase II transcription repression by the histone variant macroH2A. Mol. Cell Biol..

[31] Angelov D (2003). The histone variant macroH2A interferes with transcription factor binding and SWI/SNF nucleosome remodeling. Mol. Cell.

[32] Halder R (2016). DNA methylation changes in plasticity genes accompany the formation and maintenance of memory. Nat. Neurosci..

[33] Maddox SA, Watts CS, Schafe GE (2014). DNA methyltransferase activity is required for memory-related neural plasticity in the lateral amygdala. Neurobiol. Learn. Mem..

[34] Walters BJ, Zovkic IB (2015). Building up and knocking down: an emerging role for epigenetics and proteasomal degradation in systems consolidation. Neuroscience.

[35] Stefanelli G (2021). The histone chaperone Anp32e regulates memory formation, transcription, and dendritic morphology by regulating steady-state H2A.Z binding in neurons. Cell Rep..

[36] Gallo FT, Katche C, Morici JF, Medina JH, Weisstaub NV (2018). Immediate early genes, memory and psychiatric disorders: focus on c-Fos, Egr1 and Arc. Front. Behav. Neurosci..

[37] Collins BE, Sweatt JD, Greer CB (2019). Broad domains of histone 3 lysine 4 trimethylation are associated with transcriptional activation in CA1 neurons of the hippocampus during memory formation. Neurobiol. Learn Mem..

[38] Lavigne MD (2015). Composite macroH2A/NRF-1 nucleosomes suppress noise and generate robustness in gene expression. Cell Rep..

[39] Gamble MJ, Frizzell KM, Yang C, Krishnakumar R, Kraus WL (2010). The histone variant macroH2A1 marks repressed autosomal chromatin, but protects a subset of its target genes from silencing. Genes Dev..

[40] Creppe C, Posavec M, Douet J, Buschbeck M (2012). MacroH2A in stem cells: a story beyond gene repression. Epigenomics.

[41] Kaas GA (2013). TET1 controls CNS 5-methylcytosine hydroxylation, active DNA demethylation, gene transcription, and memory formation. Neuron.

[42] Miller CA, Campbell SL, Sweatt JD (2008). DNA methylation and histone acetylation work in concert to regulate memory formation and synaptic plasticity. Neurobiol. Learn Mem..

[43] Miller CA (2010). Cortical DNA methylation maintains remote memory. Nat. Neurosci..

[44] Miller CA, Sweatt JD (2007). Covalent modification of DNA regulates memory formation. Neuron.

[45] Duke CG, Kennedy AJ, Gavin CF, Day JJ, Sweatt JD (2017). Experience-dependent epigenomic reorganization in the hippocampus. Learn Mem..

[46] Marco A (2020). Mapping the epigenomic and transcriptomic interplay during memory formation and recall in the hippocampal engram ensemble. Nat. Neurosci..

[47] Choo JH, Kim JD, Chung JH, Stubbs L, Kim J (2006). Allele-specific deposition of macroH2A1 in imprinting control regions. Hum. Mol. Genet.

[48] Zilberman D, Coleman-Derr D, Ballinger T, Henikoff S (2008). Histone H2A.Z and DNA methylation are mutually antagonistic chromatin marks. Nature.

[49] Barzily-Rokni M (2011). Synergism between DNA methylation and macroH2A1 occupancy in epigenetic silencing of the tumor suppressor gene p16(CDKN2A). Nucleic Acids Res..

[50] Chakravarthy S (2005). Structural characterization of the histone variant macroH2A. Mol. Cell Biol..

[51] Langmead B, Salzberg SL (2012). Fast gapped-read alignment with Bowtie 2. Nat. Methods.

[52] Ramirez F, Dundar F, Diehl S, Gruning BA, Manke T (2014). deepTools: a flexible platform for exploring deep-sequencing data. Nucleic Acids Res..

[53] Shen L, Shao N, Liu X, Nestler E (2014). ngs.plot: Quick mining and visualization of next-generation sequencing data by integrating genomic databases. BMC Genomics.

[54] Stark, R. & Brown, G. DiffBind: differential binding analysis of ChIP-Seq peak data. *Bioconductor.org* (2011).

[55] Dobin A (2013). STAR: ultrafast universal RNA-seq aligner. Bioinformatics.

[56] Love MI, Huber W, Anders S (2014). Moderated estimation of fold change and dispersion for RNA-seq data with DESeq2. Genome Biol..

