# Author Correction: Histone macroH2A1 is a stronger regulator of hippocampal transcription and memory than macroH2A2 in mice

**DOI:** 10.1038/s42003-022-03515-5

**Published:** 2022-05-27

**Authors:** Gurdeep Singh, Gilda Stefanelli, Klotilda Narkaj, Mark A. Brimble, Samantha D. Creighton, Timothy A. B. McLean, Meaghan Hall, Krista A. Mitchnick, Jacqueline Zakaria, Thanh Phung, Anas Reda, Amanda M. Leonetti, Ashley Monks, Lara Ianov, Boyer D. Winters, Brandon J. Walters, Andrew M. Davidoff, Jennifer A. Mitchell, Iva B. Zovkic

**Affiliations:** 1 Department of Cell & Systems Biology, University of Toronto, Toronto, ON M5S 3G3 Canada; 2 Department of Psychology, University of Toronto Mississauga, Mississauga, ON L5L 1C6 Canada; 3 Department of Surgery, St. Jude Children’s Research Hospital, Memphis, TN 38105 USA; 4 Department of Psychology, York University, Toronto, ON M4N 3M6 Canada; 5 Civitan International Research Center, University of Alabama at Birmingham, Birmingham, AL 35233 USA; 6 Department of Psychology, University of Guelph, Guelph, ON N1G 2W1 Canada; 7 Department of Biology, University of Toronto Mississauga, Mississauga, ON L5L 1C6 Canada

**Keywords:** Neuroscience, Chromatin

Correction to: *Communications Biology* 10.1038/s42003-022-03435-4, published online 19 May 2022.

In this article the affiliation details for Andrew M. Davidoff were incorrectly given as ‘Department of Psychology, University of Toronto Mississauga, Mississauga, ON L5L 1C6 Canada’ but should have been ‘Department of Surgery, St. Jude Children’s Research Hospital, Memphis TN 38105, USA’. The original article has been corrected.

