# Supplementary material for: Histone macroH2A1 is a stronger regulator of hippocampal transcription and memory than macroH2A2 in mice
Source: Commun Biol. 2022 May 19;5:482. doi: 10.1038/s42003-022-03435-4 (PMC9120515; doi:10.1038/s42003-022-03435-4)
Supplement: Supplementary file 1 — Supplementary Information [file 42003_2022_3435_MOESM1_ESM.pdf]

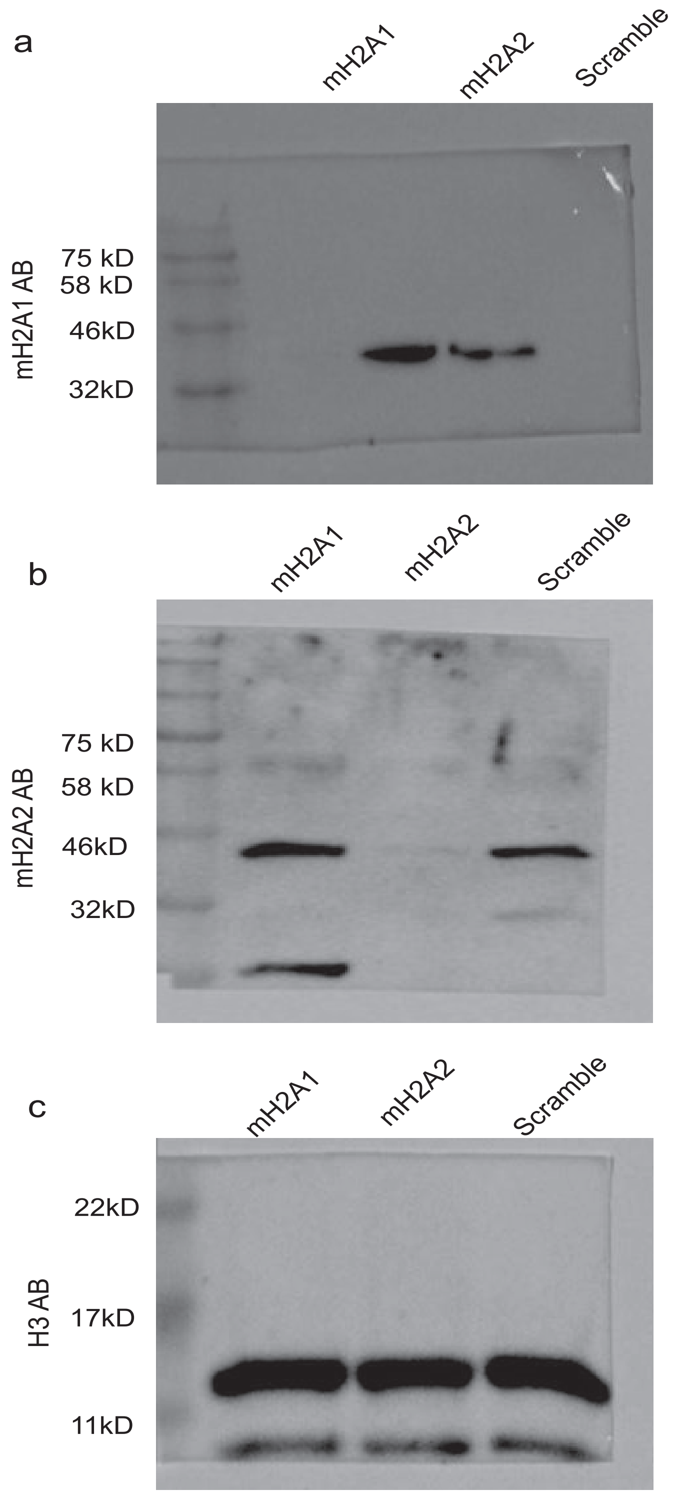

Supplementary Figure 1. Uncropped images for cropped bands shown in Figure 1A. Images show a) mH2A1, b) mH2A2, and c) H3.

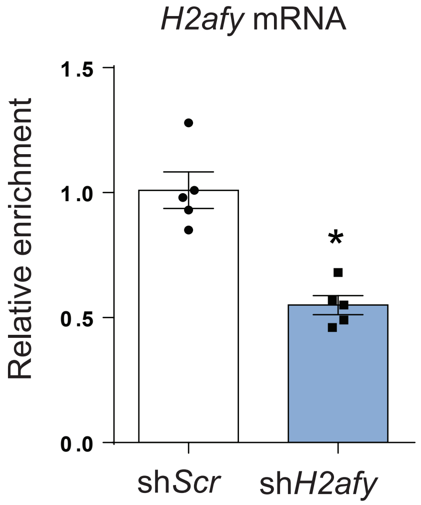

Supplementary Figure 2. Validation of knockdown over time. To ensure that knockdown efficiency did not decrease over time, *H2afy* expression was measured in mice that underwent open field testing 30 days after fear conditioning. N = 5/group. *$p<0.05$. Data are shown as mean ±SEM.

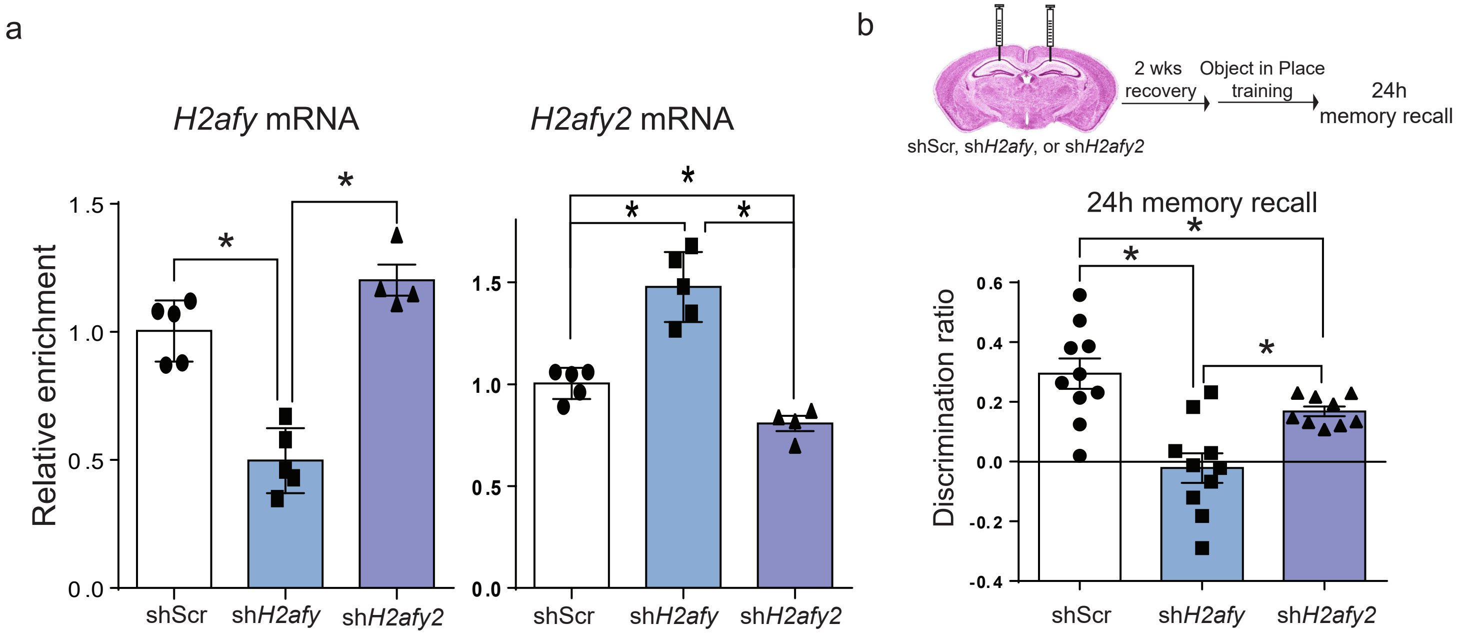

Supplementary Figure 3. Replication of viral vector-mediated effects using a second set of shRNA constructs. To ensure that behavioral effects observed with OiP were not a by-product of non-specific effects of viral constructs, we designed and packaged a new set of shRNAs against mH2A1 and mH2A2. a) Validation of knockdown in the dorsal CA1; b) Replication of OiP data using new shRNA constructs. *$p<0.05$. Data are shown as mean ±SEM.

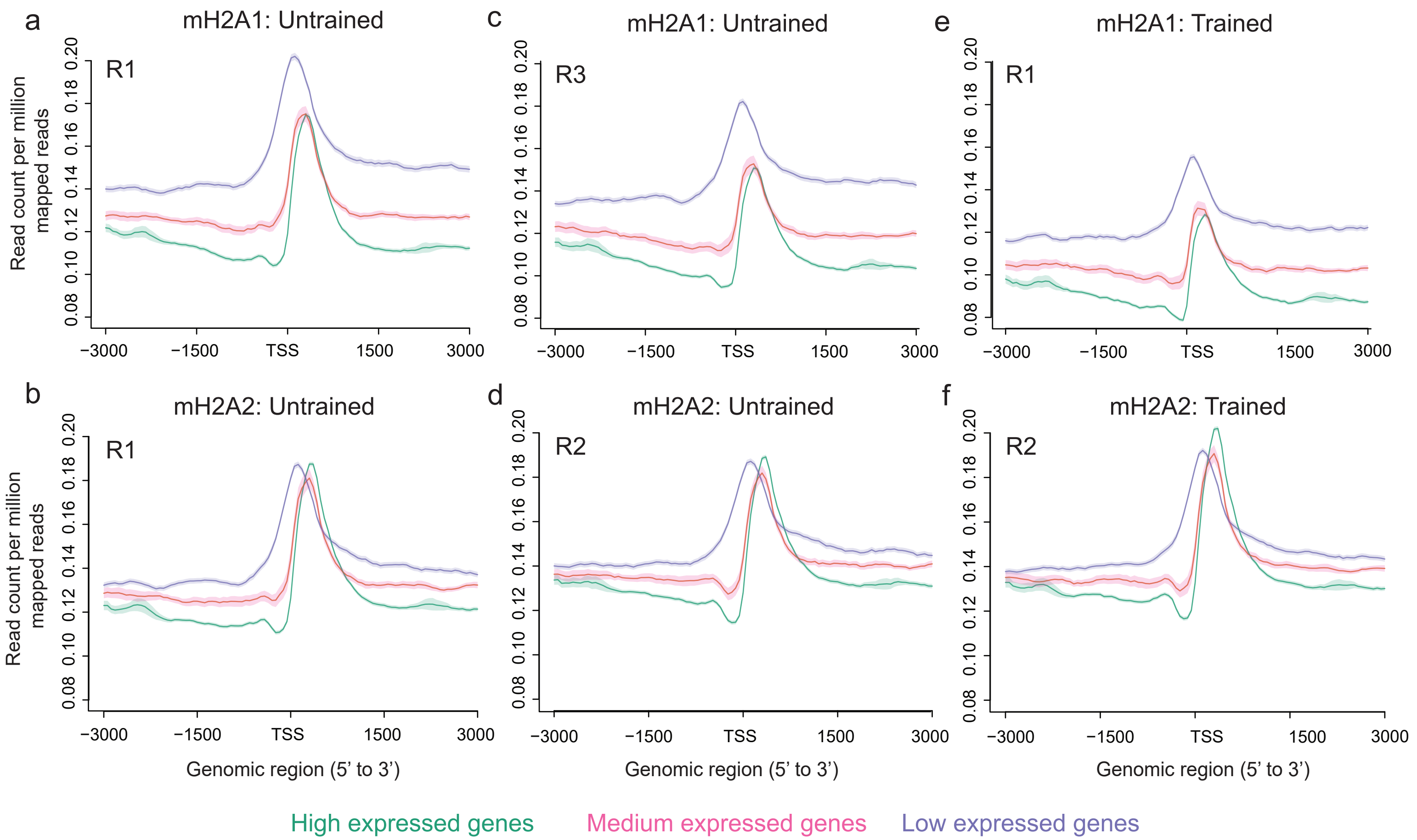

Supplementary Figure 4. mH2A binding is negatively associated with gene expression. Average profile plot of mH2A1 (a,c,e) and mH2A2 (b,d,f) binding at the TSS for all genes grouped into three categories based on levels of expression (High, Medium and Low) in untrained (a,c,b,d) and trained (e,f) mice. Data are shown for the mice that were not included in Figure 4.

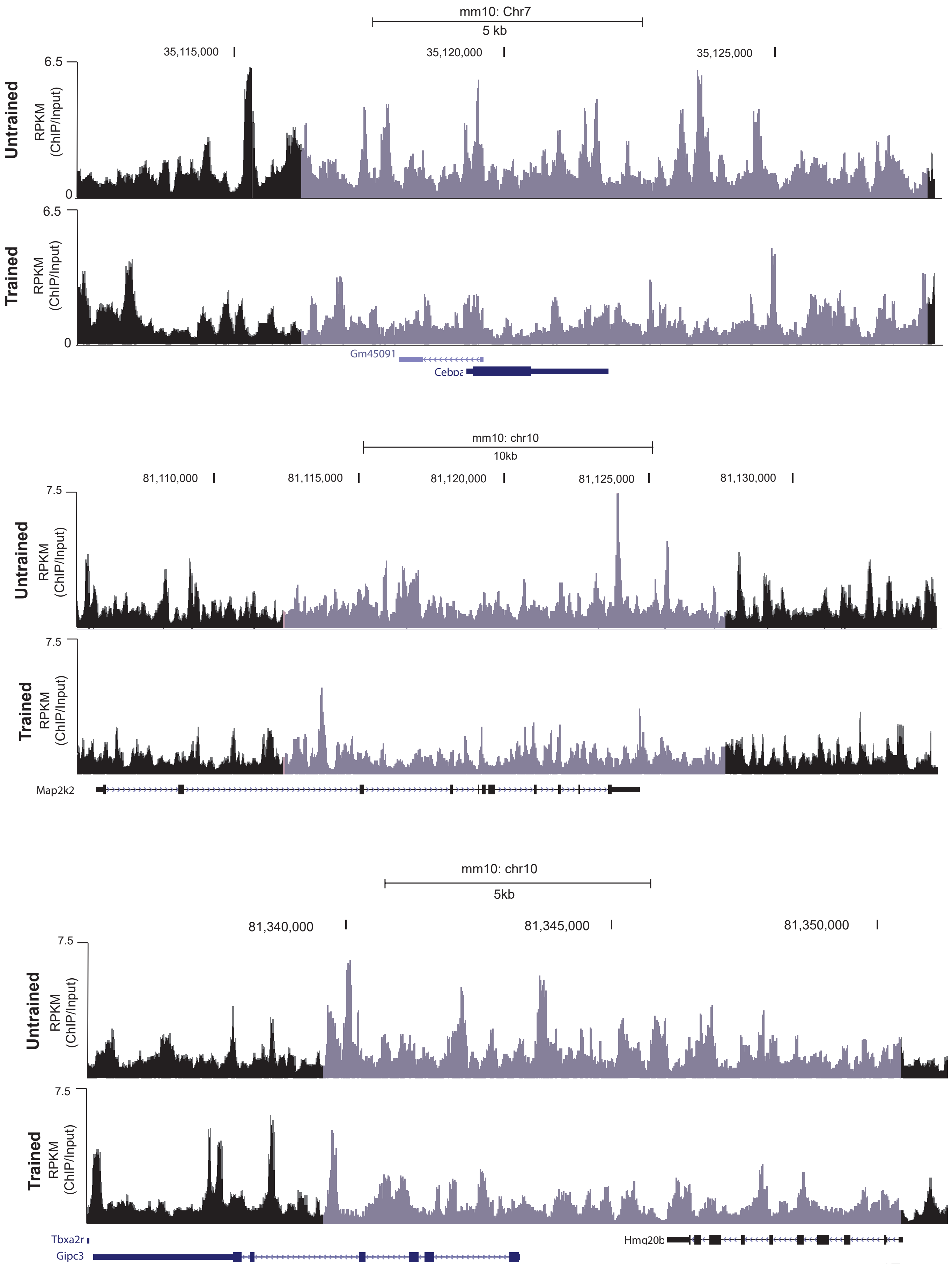

Supplementary Figure 5. Examples of genes that lost mH2A1 signal 30 min after training. Several examples of differentially bound regions (ChIP divided by Input), identified by DiffBind based on epic2 peaks are highlighted in the UCSC Genome Browser track (mm10 assembly).

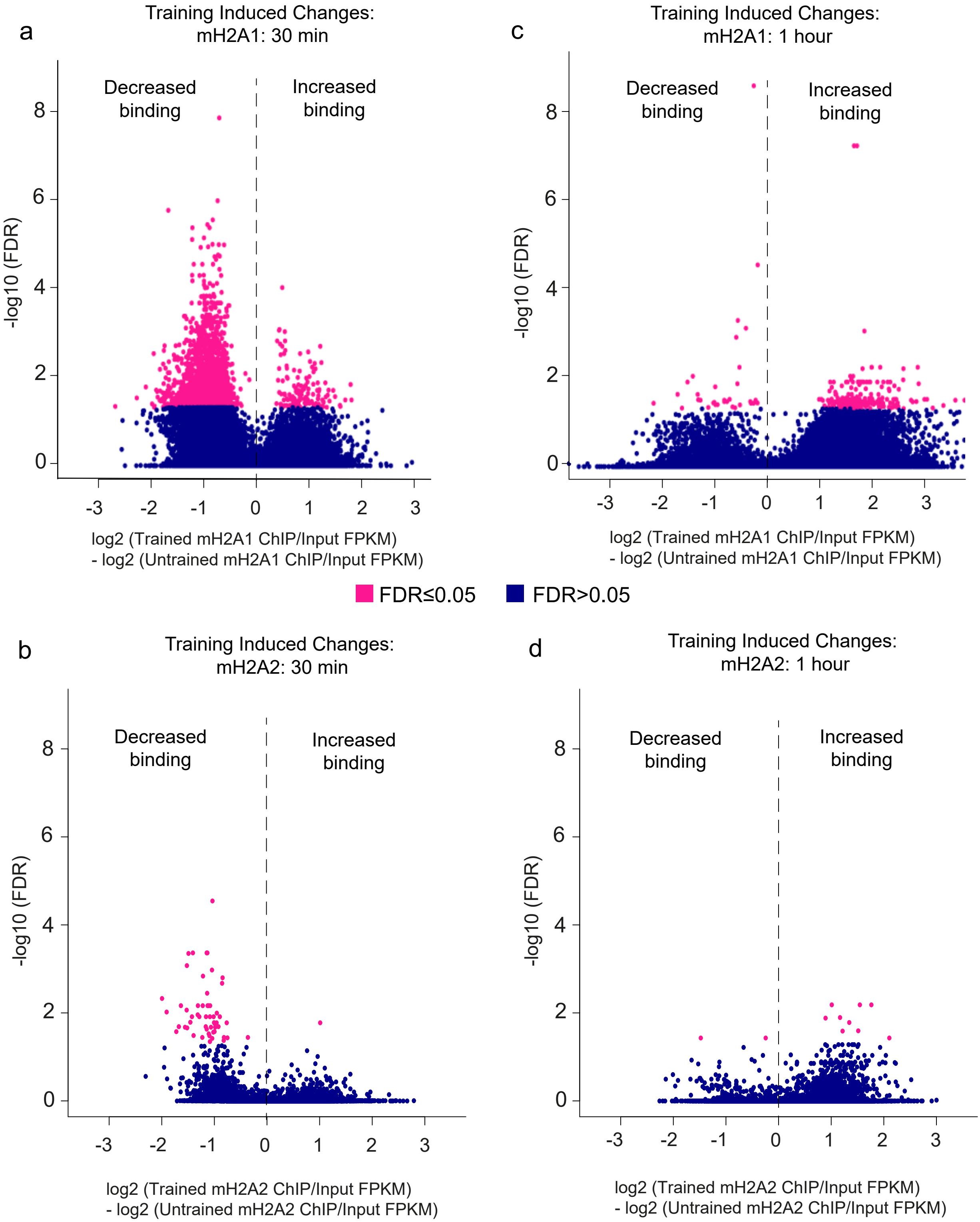

Supplementary Figure 6. Binding of mH2A1, not mH2A2, narrow peaks is dynamically regulated during learning. DiffBind analysis of mH2A1 (a) and mH2A2 (b) consensus peaks (using MACS2 peak caller) shows substantial loss of mH2A1 peaks only 30 min after fear conditioning. Changes in narrow peaks at 1h for mH2A1 (c) and mH2A2 (d)

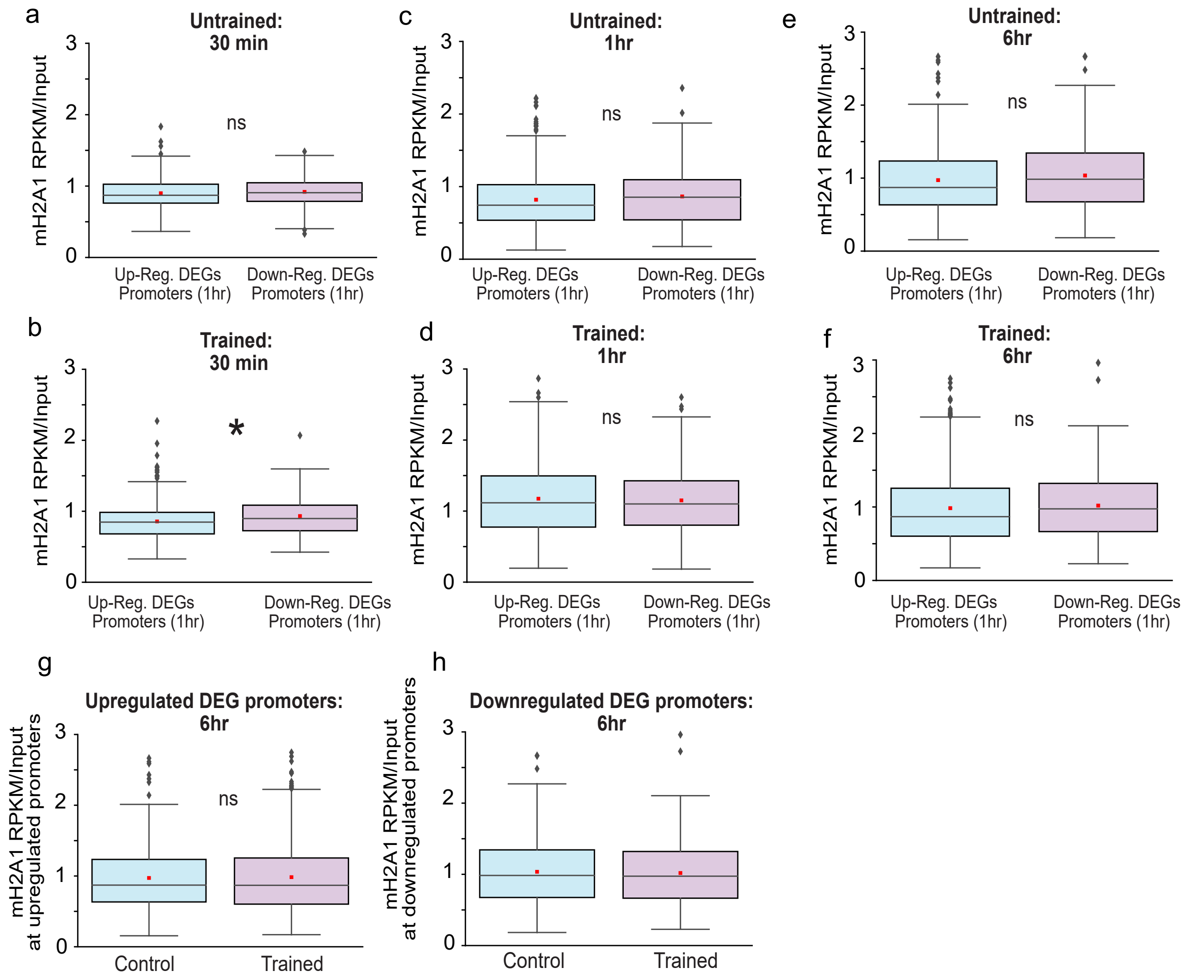

Supplementary Figure 7. mH2A1 binding divergence at promoters 30 min after fear conditioning, not at later time points, is correlated with genes that are up- and downregulated 1h after training. Box plots showing comparison of mH2A1 binding between promoters of upregulated and downregulated DEGs (RNA-seq at 1 hour) within untrained samples (a, c, e) and trained mice at three timepoints: 30 min (b), 1 hour (d) and 6 hours (f) after fear conditioning. Upregulated and downregulated DEGs which diverged in gene expression upon training show significant difference only in mH2A1 binding in the trained samples 30 min after fear conditioning, while trained mice at other higher timepoints and untrained mice show no differences. g-h) By 6 hours after fear conditioning there are no significant changes in binding of mH2A1 between trained and untrained samples at either upregulated (g) or downregulated (h) DEGs promoters.

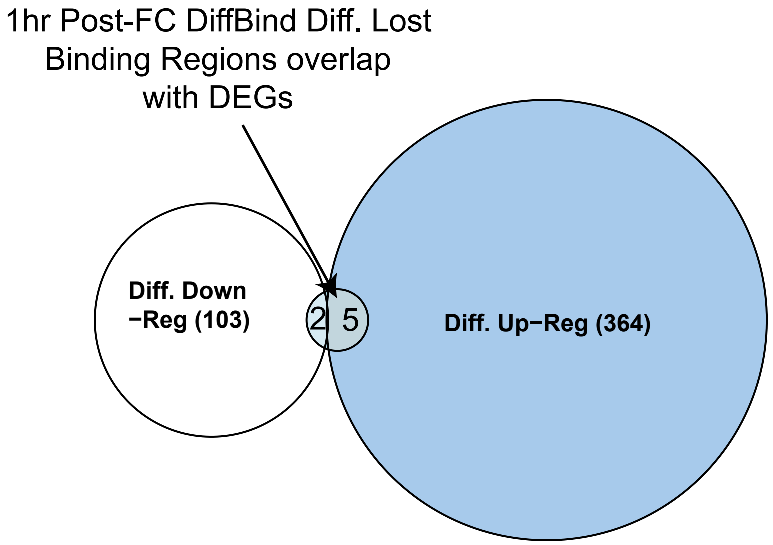

Supplementary Figure 8. Reductions in mH2A1 binding 1h after fear conditioning do not overlap with DEGs. Venn diagrams demonstrating no over-representation of reduced mH2A1 signal (within +/-100kb window) on promoters of differentially upregulated compared to downregulated DEGs

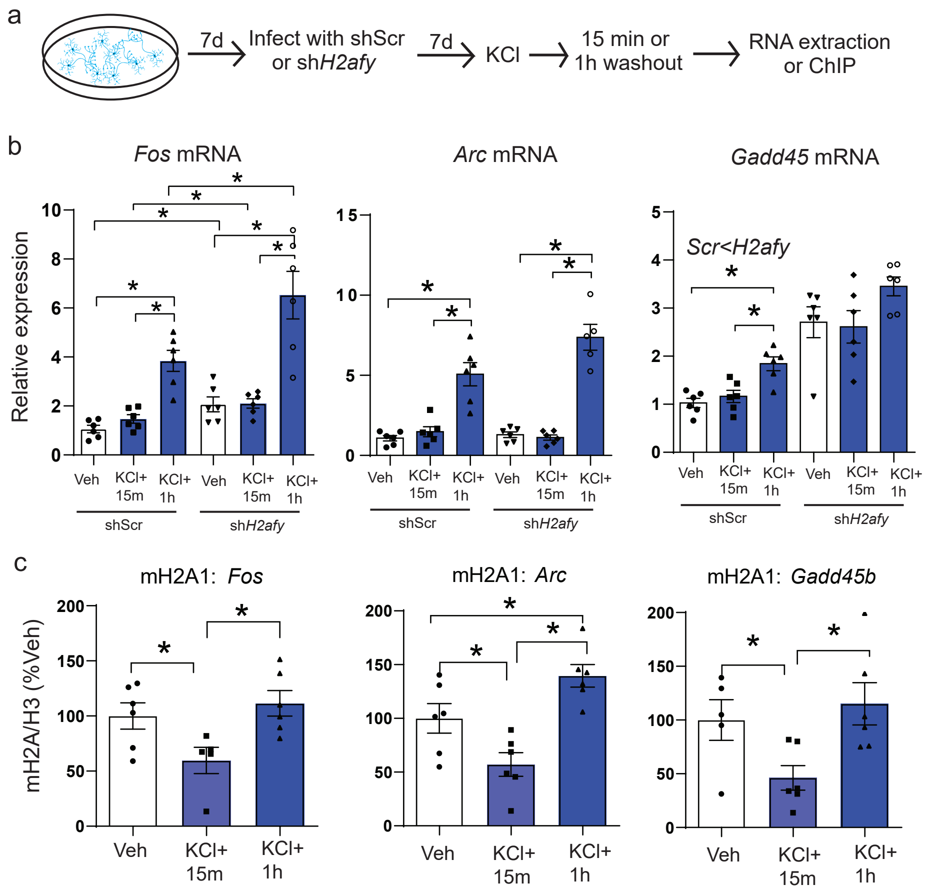

Supplementary Figure 9. mH2A1 regulates basal and activity-induced gene expression in neurons. a) Primary cortical neurons were infected with AAV vectors carrying scramble control or anti-*H2afy* shRNA at day 7 *in vitro* (DIV7). b) At DIV14, neurons were depolarized with 55mM KCl for 30 min and gene expression was measured 15 min or 1h after KCl washout in scramble control and mH2A1-deficient neurons. Scr<*H2afy*: Main effect of virus, p<0.05. c) DIV14 neurons (without AAV treatment) were depolarized with 55mM KCl for 30 min and mH2A1 binding was assessed 15 min or 1h after KCl washout. mH2A1 binding was normalized to H3 signal at the same locus. N=6 wells/group. Data are shown as mean ±SEM. *p<0.05.

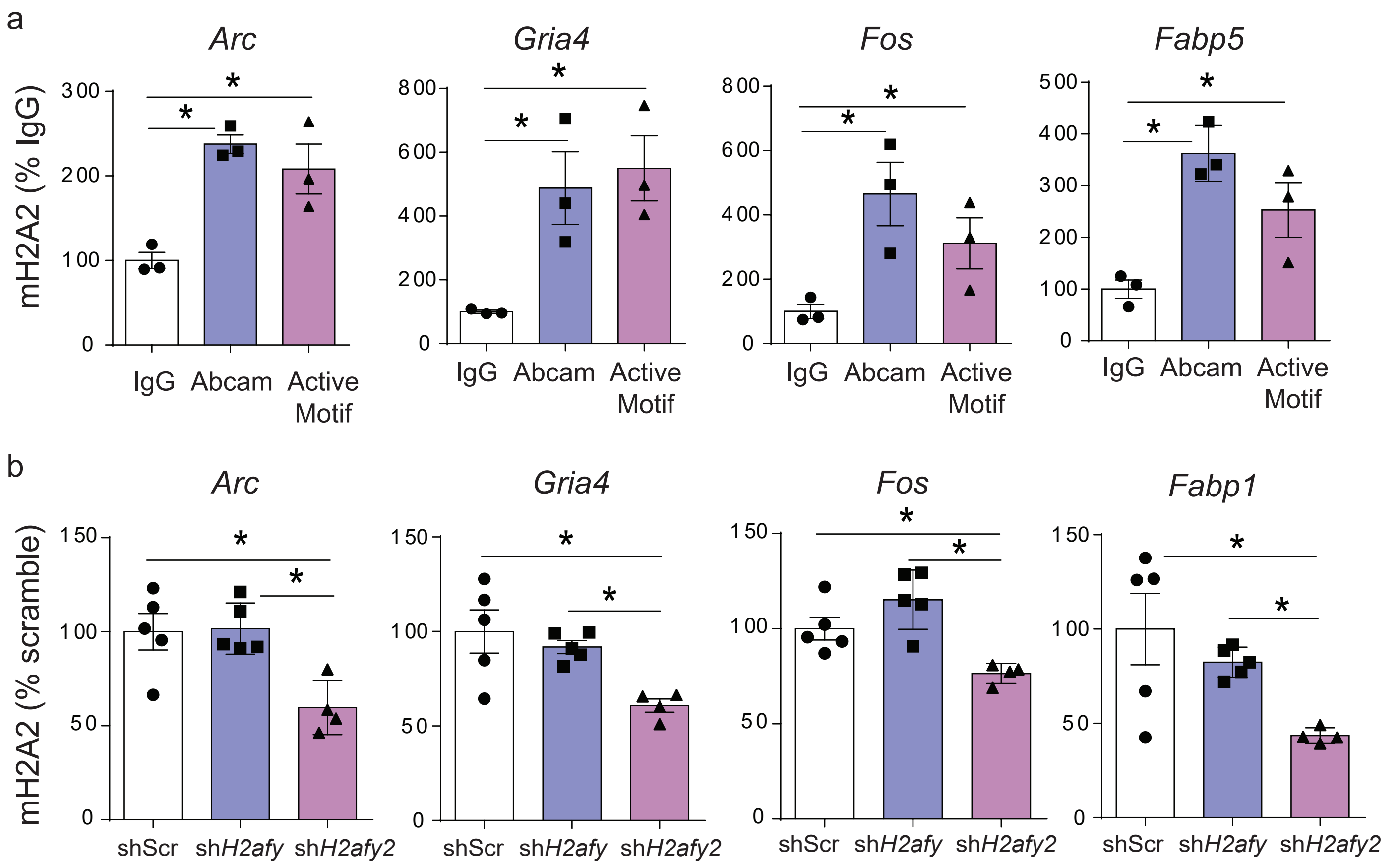

Supplementary Figure 10. Validation of mH2A2 antibody for ChIP. a) To ensure that we could achieve sufficient enrichment of ChIP signal, we compared Abcam (ab4173) antibody used here to another mH2A2 antibody from Active Motif (39873). Both resulted in enriched signal compared to IgG. N = 3/group. b) The antibody was further validated for specificity by depleting mH2A1 and mH2A2 in cultured neurons and demonstrating reduced binding only with mH2A2 depletion, thereby demonstrating antibody specificity. N = 5/group. *p<0.05. Data are shown as mean ±SEM.

**DEGs upregulated by depletion of both mH2A1 and mH2A2**
Lcn2, Ly6i, Ccl5, Ccn5, Ifi204, Ifi209, Cxcl10, Gm12250, Fcgr4, H2-Q7, Ifi27l2a H2-Q6, Gm4951, Irf7, Cd52, Oasl2, Isg15, Gimap4, Phf11d, Oas1a, Fgl2, Rsad2 ly9, Ifit1, Gbp6, Nlrc5, Ifi44, Usp18, Lgals3bp, Ifi30, Bst2, Trim30a, Gbp5, Parp14 Tap1, Ifit3, Icam1, Ddx60, Psmb8, Gbp4, Lag3, Gpnmb, Ifitm3, Rnf213, Vim, Gbp7 Parp9, C1qc, Osmr, Parp3, Igfbp2, Mmp2, Tnfrsf10b, Zc3hav1, Grn, Cdkn1a, Tap2 Ccdc88b, F2r, Scpep1, Nrros, Pm20d1, Plod2, Trp53inp1, Ly6e, Hist1h1c, Vat1 Smc4, Ephx1, Lgals8, Clcn5, Smarcd2
**DEGs downregulated by depletion of both mH2A1 and mH2A2**
Epop, Sema3a, Sowahb, Coch, Htr1b, Gm16485, Necab1, Meis2, Crhr1, Egr1 Arhgap33, Cacna1i, Hlf, Nyap2, Gucy1a1
**DEGs upregulated by depletion by mH2A1 and downregulated by mH2A2 depletion**
Fos

Supplementary Table 1. Gene ontology for differentially expressed genes in mice with hippocampal mH2A1 or mH2A2 depletion. A list of DEGs that overlap in untrained mH2A1- and mH2A2-deficient mice in Figure 2b-c, arranged by the direction of change. N=3 mice/group

Term	Overlap	P-value	Adjusted P	Odds Ratio	Combined Score
lysosomal lumen (GO:0043202)	38/86	2.10E-16	9.35E-14	4.423027679	159.6740147
secretory granule lumen (GO:0034774)	82/317	3.06E-16	6.82E-14	2.589340129	92.50205248
specific granule (GO:0042581)	53/160	9.59E-16	1.43E-13	3.315815816	114.6631687
vacuolar lumen (GO:0005775)	52/161	5.83E-15	6.50E-13	3.233046711	105.9678629
integral component of plasma membrane (GO:0005887)	237/1463	1.06E-14	9.48E-13	1.621580569	52.17466128
membrane raft (GO:0045121)	41/119	3.83E-13	2.84E-11	3.448826978	98.60897041
endoplasmic reticulum lumen (GO:0005788)	68/270	4.21E-13	2.68E-11	2.521039558	71.83866475
lysosome (GO:0005764)	90/422	2.41E-12	1.35E-10	2.134836258	57.10567244
focal adhesion (GO:0005925)	79/356	6.66E-12	3.30E-10	2.221322446	57.16672861
tertiary granule (GO:0070820)	47/164	1.44E-11	6.44E-10	2.868722381	71.60586937

Supplementary Table 2. Gene ontology for DEGs upregulated by mH2A1 depletion. Ontology is performed on RNA seq data in Figure 2b and c. N=3 mice/group

Term	Overlap	P-value	Adjusted P	Odds ratio	Combined Score
dendrite (GO:0030425)	23/215	5.11E-12	2.28E-09	6.0782241	158.0378814
integral component of plasma membrane (GO:0005887)	48/1463	2.17E-05	0.004850046	1.8641645	20.01355651
dendrite membrane (GO:0032590)	4/20	3.66E-04	0.054353908	11.363636	89.93119806
axon (GO:0030424)	9/141	8.94E-04	0.099666845	3.6266925	25.45918809
GABA-A receptor complex (GO:1902711)	3/19	0.004249	0.378976077	8.9712919	48.99368932
juxtaparanode region of axon (GO:0044224)	2/9	0.010247	0.761673553	12.626263	57.83834225
neurotransmitter receptor complex (GO:0098878)	2/12	0.018143	1	9.469697	37.96834123
main axon (GO:0044304)	3/33	0.019965	1	5.1652893	20.21568589
intrinsic component of the cytoplasmic side of the plasma membrane (GO:0031235)	2/15	0.027882	1	7.5757576	27.11963347
mitochondrial respiratory chain complex I (GO:0005747)	3/51	0.060614	1	3.342246	9.369104759

Supplementary Table 3. Gene ontology for DEGs downregulated by mH2A1 depletion. Ontology on RNA seq data presented in Figure 2b. Only the categories highlighted in blue reached statistical significance. N=3/group.
