# Supplementary material for: Histone macroH2A1 is a stronger regulator of hippocampal transcription and memory than macroH2A2 in mice
Source: Commun Biol. 2022 May 19;5:482. doi: 10.1038/s42003-022-03435-4 (PMC9120515; doi:10.1038/s42003-022-03435-4)
Supplement: Supplementary file 2 — Description of Additional Supplementary Files [file 42003_2022_3435_MOESM2_ESM.pdf]

Description of Additional Supplementary Files

File name: Supplementary Data 1.
Description: The source data behind graphs in Figure 1a

File name: Supplementary Data 2.
Description: The source data behind graphs in Figure 1b,c

File name: Supplementary Data 3.
Description: The source data behind graphs in Figure 1d

File name: Supplementary Data 4.
Description: The source data behind graphs in Figure 1e

File name: Supplementary Data 5.
Description: The source data behind graphs in Figure 6m

File name: Supplementary Data 6.
Description: Differentially expressed genes with depletion of mH2A1 or mH2A2 from Figure 2b-c

File name: Supplementary Data 7.
Description: Differentially expressed genes in response to fear conditioning in scramble control and mH2A1-depleted mice from Figure 2e-f.

File name: Supplementary Data 8.
Description: Differentially bound regions for mH2A1 and mH2A2 30 min after fear conditioning from Figure 5.

File name: Supplementary Data 9.
Description: Differentially expressed genes induced 1h after fear conditioning in intact mice without surgery from Figure 6a.

File name: Supplementary Data 10.
Description: Differentially expressed genes after fear conditioning in intact mice
