# Supplementary material for: Histone macroH2A1 is a stronger regulator of hippocampal transcription and memory than macroH2A2 in mice
Source: Commun Biol. 2022 May 19;5:482. doi: 10.1038/s42003-022-03435-4 (PMC9120515; doi:10.1038/s42003-022-03435-4)
Supplement: Supplementary file 13 — Reporting Summary [file 42003_2022_3435_MOESM13_ESM.pdf]

nature research

Corresponding author(s): Iva Zovkic
Jennifer A. Mitchell

Last updated by author(s): Mar 25, 2022

Reporting Summary

Nature Research wishes to improve the reproducibility of the work that we publish. This form provides structure for consistency and transparency in reporting. For further information on Nature Research policies, see Authors & Referees and the Editorial Policy Checklist.

Statistics

For all statistical analyses, confirm that the following items are present in the figure legend, table legend, main text, or Methods section.

n/a	Confirmed	
☐	☒	The exact sample size (n) for each experimental group/condition, given as a discrete number and unit of measurement
☐	☒	A statement on whether measurements were taken from distinct samples or whether the same sample was measured repeatedly
☐	☒	The statistical test(s) used AND whether they are one- or two-sided<br>*Only common tests should be described solely by name; describe more complex techniques in the Methods section.*
☒	☐	A description of all covariates tested
☐	☒	A description of any assumptions or corrections, such as tests of normality and adjustment for multiple comparisons
☐	☒	A full description of the statistical parameters including central tendency (e.g. means) or other basic estimates (e.g. regression coefficient) AND variation (e.g. standard deviation) or associated estimates of uncertainty (e.g. confidence intervals)
☐	☒	For null hypothesis testing, the test statistic (e.g. F, t, r) with confidence intervals, effect sizes, degrees of freedom and P value noted<br>*Give P values as exact values whenever suitable.*
☒	☐	For Bayesian analysis, information on the choice of priors and Markov chain Monte Carlo settings
☐	☒	For hierarchical and complex designs, identification of the appropriate level for tests and full reporting of outcomes
☒	☐	Estimates of effect sizes (e.g. Cohen's d, Pearson's r), indicating how they were calculated

Our web collection on statistics for biologists contains articles on many of the points above.

Software and code

Policy information about availability of computer code

Data collection	behavioral data were collected by by automated software FreezeFrame, (Coulbourn Instruments) for fear conditioning and using a camera (Microsoft LifeCam Studio) for object in place and open field.
Data analysis	Here is a list of all analyses software used for bioinformatics. For context, details are specified in the manuscript and in the GEO depository that contains our sequencing data. FastQC (v.0.11.2), Trim Galore (v.0.4.5), Cutadapt (v2.6), Bowtie2 (v2.3.4.2), [38]. multiBamSummary and plotCorrelation from deepTools, ngs.plot, DiffBind (v.2.10.0), R (v.3.5.0), RseQC package (v2.3.7), featureCounts (Subread package v ≥1.26.1), DEseq2 (v 1.22.2).<br>Percent of time spent freezing was scored by automated software FreezeFrame, Coulbourn Instruments. For open field Total time spent in the chamber was scored using EthoVision XT 8.5 (Noldus Information Technology, Wageningen, The Netherlands).

For manuscripts utilizing custom algorithms or software that are central to the research but not yet described in published literature, software must be made available to editors/reviewers. We strongly encourage code deposition in a community repository (e.g. GitHub). See the Nature Research guidelines for submitting code & software for further information.

Data

Policy information about availability of data

All manuscripts must include a data availability statement. This statement should provide the following information, where applicable:

- Accession codes, unique identifiers, or web links for publicly available datasets
- A list of figures that have associated raw data
- A description of any restrictions on data availability

The data are available on GEO accession number GSE147445.

Field-specific reporting

Please select the one below that is the best fit for your research. If you are not sure, read the appropriate sections before making your selection.

☒ Life sciences ☐ Behavioural & social sciences ☐ Ecological, evolutionary & environmental sciences

For a reference copy of the document with all sections, see nature.com/documents/nr-reporting-summary-flat.pdf

Life sciences study design

All studies must disclose on these points even when the disclosure is negative.

Sample size	Ns for behavioural studies were determined based on power analysis and our prior experience with the data.
Data exclusions	Data were excluded for outliers with z score > 2.5
Replication	There have been no replication studies.
Randomization	Mice were randomly assigned to experimental groups.
Blinding	Behavioural scoring was performed by automated software, so blinding was not an issue.

Reporting for specific materials, systems and methods

We require information from authors about some types of materials, experimental systems and methods used in many studies. Here, indicate whether each material, system or method listed is relevant to your study. If you are not sure if a list item applies to your research, read the appropriate section before selecting a response.

Materials & experimental systems

n/a	Involved in the study	
☐	☒	Antibodies
☒	☐	Eukaryotic cell lines
☒	☐	Palaeontology
☐	☒	Animals and other organisms
☒	☐	Human research participants
☒	☐	Clinical data

Methods

n/a	Involved in the study	
☐	☒	ChIP-seq
☒	☐	Flow cytometry
☒	☐	MRI-based neuroimaging

Antibodies

Antibodies used	mH2A1 (Abcam cat# ab37264 for 30 min; Thermo Fisher cat# Ma5-24696 for 1h and 6h time points due to 564 prolonged supply issue from Abcam), mH2A.2 (Abcam cat#ab4173), H3 (Cell Signaling, Cat# 2650S)
Validation	Please refer to materials and methods section "antibody validation" for an extensive validation of all the antibodies used

Animals and other organisms

Policy information about studies involving animals; ARRIVE guidelines recommended for reporting animal research

Laboratory animals	Male C57BL/6J mice (Jackson Laboratories) of approximately 9–12 weeks of age were used for the experiments.
Wild animals	N/A
Field-collected samples	N/A
Ethics oversight	All procedures were approved by the University of Toronto Animal Care Committee and performed in accordance with the Canadian Council on Animal Care guidelines.

Note that full information on the approval of the study protocol must also be provided in the manuscript.

ChIP-seq

Data deposition

☒ Confirm that both raw and final processed data have been deposited in a public database such as GEO.

☒ Confirm that you have deposited or provided access to graph files (e.g. BED files) for the called peaks.

Data access links
May remain private before publication.

The data are available on GEO accession number GSE147445.

Files in database submission

ChIP_Control_Rep1_macroH2A1.bam
ChIP_Control_Rep2_macroH2A1.bam
ChIP_Control_Rep3_macroH2A1.bam
ChIP_Control_Rep1_macroH2A2.bam
ChIP_Control_Rep2_macroH2A2.bam
ChIP_Control_Rep3_macroH2A2.bam
ChIP_ Control_Rep1_input.bam
ChIP_ Control_Rep2_input.bam
ChIP_ Control_Rep3_input.bam
ChIP_Fear conditioned_Rep1_macroH2A1.bam
ChIP_Fear conditioned_Rep2_macroH2A1.bam
ChIP_Fear conditioned_Rep1_macroH2A2.bam
ChIP_Fear conditioned_Rep2_macroH2A2.bam
ChIP_Fear conditioned_Rep1_input.bam
ChIP_Fear conditioned_Rep2_input.bam
RNA_Control_Rep1_Aligned.sortedByCoord.out.bam RNA_Control_Rep1_Aligned.sortedByCoord.out.bam.bai
RNA_Control_Rep2_Aligned.sortedByCoord.out.bam RNA_Control_Rep2_Aligned.sortedByCoord.out.bam.bai
RNA_Control_Rep3_Aligned.sortedByCoord.out.bam RNA_Control_Rep3_Aligned.sortedByCoord.out.bam.bai
RNA_Fear conditioned_Rep1_Aligned.sortedByCoord.out.bam
RNA_Fear conditioned_Rep2_Aligned.sortedByCoord.out.bam

Rep1_shH2afy_1.fastq.gz Rep1_shH2afy_2.fastq.gz
Rep2_shH2afy_1.fastq.gz Rep2_shH2afy_2.fastq.gz
Rep3_shH2afy_1.fastq.gz Rep3_shH2afy_2.fastq.gz
Rep1_shH2afy_1.fastq.gz Rep1_shH2afy_2.fastq.gz
Rep2_shH2afy_1.fastq.gz Rep2_shH2afy_2.fastq.gz
Rep3_shH2afy_1.fastq.gz Rep3_shH2afy_2.fastq.gz
Rep1_shScramble_1.fastq.gz Rep1_shScramble_2.fastq.gz
Rep2_shScramble_1.fastq.gz Rep2_shScramble_2.fastq.gz
Rep3_shScramble_1.fastq.gz Rep3_shScramble_2.fastq.gz

Control_Rep1_mH2A1.bed bed
Control_Rep2_mH2A1.bed bed
Control_Rep3_mH2A1.bed bed
Control_Rep1_mH2A2.bed bed
Control_Rep2_mH2A2.bed bed
Control_Rep3_mH2A2.bed bed
Fear conditioned_Rep1_mH2A1.bed bed
Fear conditioned_Rep2_mH2A1.bed bed
Fear conditioned_Rep1_mH2A2.bed bed
Fear conditioned_Rep2_mH2A2.bed bed
Fear conditioning_RNA_seq_Raw_Counts.csv csv
mH2A1_KD_RNA_seq_Raw_Counts.csv csv
mH2A2_KD_RNA_seq_Raw_Counts.csv csv
Scramble_KD_RNA_seq_Raw_Counts.csv csv
SSCR13_N_S1_MLRall_R1_001.fastq.gz
SCR13_N_S1_MLRall_R2_001.fastq.gz
SCR14_N_S2_MLRall_R1_001.fastq.gz
SCR14_N_S2_MLRall_R2_001.fastq.gz
SCR19_1h_S3_MLRall_R1_001.fastq.gz
SCR19_1h_S3_MLRall_R2_001.fastq.gz
SCR20_1h_S4_MLRall_R1_001.fastq.gz
SCR20_1h_S4_MLRall_R2_001.fastq.gz
SCR23_1h_S5_MLRall_R1_001.fastq.gz
SCR23_1h_S5_MLRall_R2_001.fastq.gz
SCR24_N_S6_MLRall_R1_001.fastq.gz
SCR24_N_S6_MLRall_R2_001.fastq.gz
Y13_N_S7_MLRall_R1_001.fastq.gz
Y13_N_S7_MLRall_R2_001.fastq.gz
Y14_N_S8_MLRall_R1_001.fastq.gz
Y14_N_S8_MLRall_R2_001.fastq.gz

Y19_1h_S9_MLRall_R1_001.fastq.gz
Y19_1h_S9_MLRall_R2_001.fastq.gz
Y20_1h_S10_MLRall_R1_001.fastq.gz
Y20_1h_S10_MLRall_R2_001.fastq.gz
Y23_1h_S11_MLRall_R1_001.fastq.gz
Y23_1h_S11_MLRall_R2_001.fastq.gz
Y24_N_S12_MLRall_R1_001.fastq.gz
Y24_N_S12_MLRall_R2_001.fastq.gz

RNA_seq_FC_N_KD.txt

Genome browser session (e.g. UCSC)

N/A

Methodology

Replicates

All sequencing data used 3 biological replicates for the untested control group and 2 biological replicates for the trained/fear conditioned group. For viral vector mediated depletion studies, 3 mice were used in each of the 3 conditions (shScramble, shH2afy, shH2afy2)

Sequencing depth

~50 million total 100 base-pair paired end reads

Antibodies

mH2A1 (Abcam cat# ab37264 for 30 min; Thermo Fisher Ma5-24696 for 1h and 6h), mH2A.2 (Abcam cat#ab4173)

Peak calling parameters

Broad and diffusely enriched domains (regions enriched for reads compared to input) were identified using epic2 (v 0.0.39; FDR < 0.05) and MACS2 (v 2.2.7.1; p value cutoff = 5e-3; broad-cutoff as 0.05 or 0.01)

Data quality

Quality was assessed using FastQC (v.0.11.2) and adaptors were trimmed using Trim Galore (v.0.4.5) running Cutadapt (v2.6) and only paired reads were retained in the analysis. Reads were aligned to the UCSC Mus Musculus mm10 reference genome using Bowtie2 (v2.3.4.2) [38]. multiBamSummary and plotCorrelation from deepTools [39] was used for correlation analysis of mH2A1 and mH2A2 binding. Heatmap and average profile plots giving mean read counts per million mapped reads for all specified regions at TSSs were generated using ngs.plot [40]. Peaks (regions enriched for reads) were identified using epic2 and MACS2 and consensus peaks for all samples were assembled based on peak presence in at least 2 samples. Differential analyses for mH2A1 and mH2A2 binding in Untrained and Trained conditions were carried out with using DiffBind (v.2.10.0) [41] with R (v.3.5.0) and significance was set at FDR < 0.05. Peaks from epic2 for each sample along with respective bam files for ChIP experiment and input control under respective training conditions were used as input for DiffBind which uses DESeq2 package for differential analysis. For the statistical tests of mH2A binding at DEGs, the aligned bam files for each sample were merged under respective trained or untrained condition. mH2A binding RPKM reads at promoters were normalized using the respective input control reads.

For mH2A1: At FDR 5%, we have 2316 epic2 peaks which lost mH2A1 and 126 peaks which gained mH2A1. The individual values are shown in the attached excel sheet, which shows fold change in log values. Thus, 5-fold change will be equivalent to log2(5) = 2.322.As such, 1551 peaks lost mH2A1 (these are -2.34 to -9.53 in excel sheet) and 41 peaks gained mH2A1 (these are +2.42 to +6.47 in excel sheet).
For mH2A2: At FDR 5%, 5 peaks lost mH2A2 and 2 peaks gained mH2A2, as specified in the text. Of these, 2 peaks which lost mH2A2 and 2 peaks which gained mH2A2 were at 5 fold change or above

Software

For context, details are specified in the manuscript and in the GEO depository that contains our sequencing data. FastQC (v.0.11.2), Trim Galore (v.0.4.5), Cutadapt (v2.6), Bowtie2 (v2.3.4.2), [38]. multiBamSummary and plotCorrelation from deepTools, ngs.plot, DiffBind (v.2.10.0), R (v.3.5.0)
